# Changes in Intake of Fruits and Vegetables and Weight Change in United States Men and Women Followed for Up to 24 Years: Analysis from Three Prospective Cohort Studies

**DOI:** 10.1371/journal.pmed.1001878

**Published:** 2015-09-22

**Authors:** Monica L. Bertoia, Kenneth J. Mukamal, Leah E. Cahill, Tao Hou, David S. Ludwig, Dariush Mozaffarian, Walter C. Willett, Frank B. Hu, Eric B. Rimm

**Affiliations:** 1 Department of Nutrition, Harvard T. H. Chan School of Public Health, Boston, Massachusetts, United States of America; 2 Channing Division of Network Medicine, Department of Medicine, Brigham & Women’s Hospital, Harvard Medical School, Boston, Massachusetts, United States of America; 3 Department of Medicine, Beth Israel Deaconess Medical Center, Boston, Massachusetts, United States of America; 4 Department of Epidemiology, Harvard T. H. Chan School of Public Health, Boston, Massachusetts, United States of America; 5 New Balance Foundation Obesity Prevention Center, Boston Children's Hospital, Boston, Massachusetts, United States of America; 6 Division of Cardiovascular Medicine, Brigham and Women’s Hospital and Harvard Medical School, Boston, Massachusetts, United States of America; 7 Friedman School of Nutrition Science and Policy, Tufts University, Boston, Massachusetts, United States of America; Harvard University, UNITED STATES

## Abstract

**Background:**

Current dietary guidelines recommend eating a variety of fruits and vegetables. However, based on nutrient composition, some particular fruits and vegetables may be more or less beneficial for maintaining or achieving a healthy weight. We hypothesized that greater consumption of fruits and vegetables with a higher fiber content or lower glycemic load would be more strongly associated with a healthy weight.

**Methods and Findings:**

We examined the association between change in intake of specific fruits and vegetables and change in weight in three large, prospective cohorts of 133,468 United States men and women. From 1986 to 2010, these associations were examined within multiple 4-y time intervals, adjusting for simultaneous changes in other lifestyle factors, including other aspects of diet, smoking status, and physical activity. Results were combined using a random effects meta-analysis. Increased intake of fruits was inversely associated with 4-y weight change: total fruits -0.53 lb per daily serving (95% CI -0.61, -0.44), berries -1.11 lb (95% CI -1.45, -0.78), and apples/pears -1.24 lb (95% CI -1.62, -0.86). Increased intake of several vegetables was also inversely associated with weight change: total vegetables -0.25 lb per daily serving (95% CI -0.35, -0.14), tofu/soy -2.47 lb (95% CI, -3.09 to -1.85 lb) and cauliflower -1.37 lb (95% CI -2.27, -0.47). On the other hand, increased intake of starchy vegetables, including corn, peas, and potatoes, was associated with weight gain. Vegetables having both higher fiber and lower glycemic load were more strongly inversely associated with weight change compared with lower-fiber, higher-glycemic-load vegetables (*p* < 0.0001). Despite the measurement of key confounders in our analyses, the potential for residual confounding cannot be ruled out, and although our food frequency questionnaire specified portion size, the assessment of diet using any method will have measurement error.

**Conclusions:**

Increased consumption of fruits and non-starchy vegetables is inversely associated with weight change, with important differences by type suggesting that other characteristics of these foods influence the magnitude of their association with weight change.

## Abstract 2

Using longitudinal data from health practitioners, Bertoia and colleagues explore associations between specific food choices and weight change.

## Editors' Summary

**Background:**

Obesity—having an unhealthy amount of body fat—is increasing worldwide. In the United States, for example, more than a third of adults are obese and another third are overweight. Obesity is defined as having a body mass index (BMI; an indicator of body fat calculated by dividing a person’s weight in kilograms by their height in meters squared) of more than 30 kg/m^2^; overweight individuals have a BMI of 25.0–29.9 kg/m^2^. Compared to people with a healthy weight, overweight and obese individuals have an increased risk of developing diabetes and cardiovascular diseases (conditions that affect the heart and/or the blood vessels), and tend to die younger. People gain too much fat by consuming food and drink that contains more energy (calories) than they need for their daily activities. So, people can avoid becoming obese or reduce their BMI by eating a healthy diet that contains fewer calories and by exercising more.

**Why Was This Study Done?:**

The 2010 Dietary Guidelines for Americans recommend that adults and children should eat a variety of fruits and vegetables to help them achieve and maintain a healthy weight. But are all fruits and vegetables equally good at controlling weight? Fruits and vegetables differ in their dietary fiber content and their glycemic load. High fiber foods increase satiety (feeling full after eating), which can reduce total energy intake. Foods with a low glycemic load produce smaller and fewer blood sugar spikes after they are consumed, which may reduce hunger later on. In this study, the researchers investigate whether consumption of fruits and vegetable with a higher fiber content or lower glycemic load is more strongly associated with a healthy weight than consumption of fruits and vegetables with a lower fiber content or higher glycemic load by analyzing data on weight and diet changes among US men and women enrolled in three large prospective cohort studies set up to examine risk factors for major chronic diseases.

**What Did the Researchers Do and Find?:**

The researchers examined associations between changes in the intake of specific fruits and vegetables recorded in dietary questionnaires completed every 4 y and self-reported weight changes in 133,468 US men and women followed for up to 24 y. After adjusting for self-reported changes in other lifestyle factors likely to affect weight, such as smoking status and physical activity, an increased intake of fruits and of several vegetables was inversely associated with 4-y weight change. Thus, an increase in total fruit intake was associated with a change in weight over a 4-y interval of -0.53 lb (a weight loss of 0.24 kg) for each extra daily serving, and an increase in total vegetable intake was associated with a weight change of -0.25 lb (-0.11 kg) for each extra daily serving. However, increased intake of starchy vegetables such as corn, peas, and potatoes was associated with weight gain. Notably, higher-fiber, lower-glycemic load vegetables (for example, broccoli and Brussels sprouts) were more strongly inversely associated with weight change than lower-fiber, higher-glycemic load vegetables (for example, carrots and cabbage).

**What Do These Findings Mean?:**

These findings suggest that increased consumption of fruits and non-starchy vegetables is inversely associated with weight change and that different fruits and vegetables have different effects on weight. The benefits of increased consumption were greater for fruits than for vegetables and strongest for berries, apples/pears, tofu/soy, cauliflower, and cruciferous and green leafy vegetables. Increased satiety with fewer calories could be partly responsible for the beneficial effects of increasing fruit and vegetable intake. These findings may not be generalizable—nearly all the participants were well-educated white adults. Moreover, the use of dietary questionnaires and self-reported weight measurement may have introduced measurement errors into this study and, although the researchers accounted for some key lifestyle factors that are likely to affect weight, individuals who increased their fruit and vegetable intake and lost weight may have shared other unknown characteristics that were actually responsible for their weight loss. Overall, however, these findings provide new food-specific guidance for the prevention of obesity, a primary risk factor for many life-shortening health conditions.

**Additional Information:**

This list of resources contains links that can be accessed when viewing the PDF on a device or via the online version of the article at http://dx.doi.org/10.1371/journal.pmed.1001878.

- The World Health Organization provides information on obesity (in several languages)
- The Global Burden of Disease websitey provides the latest details about global obesity trends; the International Obesity Taskforce also provides information about the global obesity epidemic
- The United Kingdom National Health Service Choices website provides information about obesity, cardiovascular disease, and diabetes (including some personal stories), and about healthy eating
- The American Heart Association provides information on cardiovascular disease and diabetes and on keeping healthy, including nutritional information
- The US Centers for Disease Control and Prevention has information on all aspects of overweight and obesity
- ChooseMyPlate.gov is a resource provided by the US Department of Agriculture that provides individuals and healthcare professionals with user-friendly nutritional information; the 2010 Dietary Guidelines for Americans are available
- MedlinePlus provides links to other sources of information on obesity, heart disease, vascular disease, and diabetes (in English and Spanish)
- More information about the three cohort studies that provided data for this analysis (Nurses' Health Study and Nurses' Health Study II and Health Professionals Follow-up Study) is available

## Introduction

The 2010 Dietary Guidelines for Americans recommends eating a variety of fruits and vegetables to lower risk of chronic disease and to “help adults and children achieve and maintain a healthy weight” [1]. This guidance has a strong evidence base for the prevention of cardiovascular disease, but less so for maintaining a healthy weight. Recently, we reported associations between increased total fruit and total vegetable consumption and weight change in three separate, large prospective studies of 120,877 United States men and women aged 30–65 y at baseline [2]. However, different fruits and vegetables have individual characteristics that may impact their effects on satiety, glycemic and insulinemic responses, total calorie intake, or energy expenditure. How they are consumed may also influence these factors, for example, preparation method, portion size, complements, and substitutes.

Components of fruits and vegetables that may differentiate their impact on weight change include fiber content, glycemic load (GL), and biologically active constituents like polyphenols and sugars. Higher fiber intake increases satiety, which in turn may reduce total energy intake and prevent weight gain [3–7]. Also, lower-GL foods produce fewer and smaller postprandial glucose spikes that may decrease subsequent hunger and reduce total energy intake [8]. Furthermore, clinical trial evidence suggests that low-GL or low-glycemic-index (GI) diets may increase resting energy expenditure [9], promoting weight maintenance. In addition, polyphenols, found in meaningful concentrations in many fruits and vegetables, may influence insulin sensitivity [10], the gut microbiome [11], or the anabolic state of adipose tissue, which over a long period of time could promote relative weight stability.

The objective of this study was to examine the relationship between increased fruit and vegetable consumption and weight change over time, including subtypes and individual fruits and vegetables. We limit our analyses to whole fruits, as fruit juice typically includes several grams of added sugars and is associated with an increased risk of diabetes [12] and greater weight gain [2].

## Methods

The study protocol was approved by the Institutional Review Board of the Brigham and Women's Hospital and by the Harvard School of Public Health Human Subjects Committee Review Board (ID 2008P000327). All participants provided voluntary responses to mailed questionnaires that served as the participants’ informed consent and research aims and use of data were fully explained to each participant.

### Study Design and Population

The study population includes three prospective cohorts of men and women. The Nurses’ Health Study (NHS) is a cohort of 121,701 female nurses from 11 US states aged 30–55 y at enrollment in 1976 [13]. The Health Professionals Follow-up Study (HPFS) is a parallel cohort of 51,529 male health professionals from 50 states aged 40–75 y at enrollment in 1986 [14]. The Nurses’ Health Study II (NHS II) is a cohort of 116,686 younger female nurses aged 25–42 y at enrollment in 1989 from 14 states [15]. Men in the HPFS contributed an average of 3.3 4-y intervals and women in the NHS and NHS II 3.4 4-y intervals. Ninety-nine percent of men in the HPFS are white, 97% of women in the NHS, and 99% of women in the NHS II.

We excluded men and women with a history of chronic disease at baseline, including those who had a history of diabetes, cancer, cardiovascular disease, renal disease, pulmonary disease, liver disease, ulcerative colitis, lupus, tuberculosis, multiple sclerosis, amyotrophic lateral sclerosis, or Parkinson’s disease at baseline. We censored individuals who developed these conditions during follow-up: at time of diagnosis for cardiovascular disease and 6 y prior for all other diseases. We also excluded individuals who had gastric bypass surgery and newly pregnant or lactating women (one 4-y interval only) and censored individuals at age 65 due to age-related loss of lean muscle mass. Finally, we excluded men and women who had missing baseline lifestyle habits data, who reported implausible energy intake, or who had blank responses for more than 70 items on the food frequency questionnaire (FFQ). We defined implausible energy intake as <800 or >4,200 calories for men and <600 or >3,500 calories for women. After exclusions, 35,408 women in the NHS, 17,996 men in the HPFS, and 64,514 women in the NHS II were included in our analysis (details in S18 Table).

### Weight Change

Participants in all three cohorts reported height in inches at enrollment and current weight in pounds on biennial questionnaires. Weight change was calculated as the difference in weight between the beginning and end of each 4-y interval, therefore positive differences represent weight gain, and negative differences weight loss. Although these measures are self-reported, they are shown to be valid in these cohorts: among a sample of 123 men in the HPFS and 140 women in the NHS, Pearson correlations coefficients between self-reported weight and technician-measured weight were 0.97 [16].

### Dietary Assessment

A validated [17] 131-item semi-quantitative FFQ was administered every 4 y beginning in 1986 in the NHS and HPFS, and in 1991 in the NHS II. We included all fruits and all vegetables on the FFQ in our analyses (S7 Table). Fruits and vegetables with similar nutritional value, including fiber and GL, were combined, for example, apples and pears. We had a total of six 4-y time intervals in the NHS and HPFS (1986–2010, 24 y) and four 4-y time intervals in the NHS II (1991–2007, 16 y). The Harvard University food composition database, derived from the US Department of Agriculture (USDA) data and other outside published sources, was used to calculate nutrients consumed from food items. The USDA defines potatoes as a vegetable; however, most Americans do not consider french fries and potato chips a healthy choice. Therefore, we used unprocessed potatoes for our main analysis (baked, boiled, or mashed white potatoes, sweet potatoes, and yams) and included fried potatoes (french fries and potato chips) as a covariate. This distinction is consistent with previous work [2].

We categorized fruits and vegetables as high or low fiber, defined using the median grams of fiber per serving of those fruits and vegetables included on the FFQ (1.7 grams per serving, S2 and S3 Tables). We categorized fruits and vegetables as high or low GL similarly, with cutoffs of 0.7 for vegetables and 6.5 for fruits (S4 and S5 Tables). GL was calculated by multiplying the carbohydrate content of each fruit/vegetable (grams per serving) by the glycemic index of that fruit/vegetable. In addition, we grouped fruits into categories of citrus, melon, and berries and vegetables into categories of cruciferous, green leafy, and legumes based on similar nutritional content (S6 Table). The average Pearson correlation coefficients comparing diet assessment from our FFQ with multiple 7-d food records for 55 foods was 0.48 [18], with a range 0.24 to 0.76 for individual fruits and 0.13 to 0.53 for individual vegetables (S17 Table) [19].

### Covariates

Biennial questionnaires additionally asked participants to report lifestyle habits and any recent physician-diagnosed diseases. We included the following individual-level covariates in all models: baseline age and body mass index (BMI) for that particular time interval; change in the following lifestyle variables over the same time interval: smoking status, physical activity level [20], hours of sitting or watching TV, and hours of sleep; and change in intake of the following foods and nutrients: fried potatoes, juice, whole grains, refined grains, fried foods, nuts, whole-fat dairy, low-fat dairy, sugar-sweetened beverages, diet beverages, sweets, processed meats, non-processed meats, *trans* fat, alcohol, and seafood. Total energy intake, hypertension, hypercholesterolemia, and related medications were not included as covariates because they are potentially on the causal pathway or are consequences of fruit and vegetable intake and weight change. The frequency of data collection for physical activity, hours of watching TV, and hours of sleep data varied by cohort (S1 Table).

### Statistical Analysis

Multivariable generalized linear regression models were used to examine the independent association between change in weight (lb) over 4 y and change in intake of fruits and vegetables (servings/day) over the same 4-y time interval, as described in a previous publication [2]. Because each individual contributes multiple time intervals, we used robust variance to account for within-individual repeated measures, and results are averaged across all 4-y time intervals. Analyses of total fruits and total vegetables included both variables together in one model. Fiber analyses included all fiber variables in one model: change in intake of high-fiber fruits, low-fiber fruits, high-fiber vegetables, and low-fiber vegetables; likewise for GL analyses. Fruit and vegetable subgroup analyses included all six subgroup variables in one model, and analyses of individual fruits and vegetables included all specific fruit and vegetable variables in a single model.

Change in weight and change in intake of fruits and vegetables were truncated at the 0.5th and 99.5th percentiles to minimize the influence of outliers. Missing indicators were used for categorical variables, and the last observation was carried forward for missing values of continuous variables with the exception of diet (main exposure) and weight (main outcome). Missing values were carried forward only once for diet and weight, after which the follow-up was censored. As a sensitivity analysis, we examined change in diet over 4 y and change in weight over the following 4-y interval (for example, change in diet 1986 to 1990 and change in weight 1990 to 1994). Results from the 3 cohorts were pooled using DerSimonian-Laird estimators and the Q statistic to test for heterogeneity. The 3 studies are weighted by the inverse of the sum of the study-specific variance plus the common between-studies variance (random effects pooling). All analyses used SAS version 9.2 (SAS Institute) and a two-tailed alpha of 0.05.

## Results

At baseline, men in the HPFS were an average of 47 y old, women in the NHS 49 y old, and women in the NHS II 36 y old (Table 1). After exclusions, the remaining men in the HPFS had an average BMI of 25.1 kg/m^2^, women in the NHS 24.7 kg/m^2^, and women in the NHS II 24.2 kg/m^2^ at baseline. Within each 4-y time interval, men in the HPFS gained an average of 2.1 lb, women in the NHS 2.8 lb, and women in the NHS II 5.0 lb. Men and women in all three cohorts reported a variety of fruit and vegetable intake (S19 Table).

**Table 1:** Baseline (mean, standard deviation [SD]) characteristics and average 4-y lifestyle changes (mean and 1st to 99th percentile range) of men and women in three prospective cohorts.

	HPFS		NHS		NHS II	
	*n* = 19,316		*n* = 40,415		*n* = 73,737	
	Baseline (1986)	4-y Change	Baseline (1986)	4-y Change	Baseline (1991)	4-y Change
**Age (years)**	47.0 (3.0)		48.7 (2.4)		36.4 (3.8)	
**BMI (kg/m** ^**2**^ **)**	25.1 (1.8)		24.7 (2.1)		24.2 (4.3)	
**Weight (lb)**	177 (15)	2.1 (-12.0 to 17.0)	147 (14)	2.8 (-13.5 to 21.0)	145 (27)	5.0 (-10.5 to 30.0)
**Physical activity (MET-hr/wk)**	22.9 (19.6)	5.2 (-28.6 to 78.8)	14.4 (9.7)	-1.0 (-50.4 to 40.6)	20.7 (23.8)	0.4 (-22.3 to 21.6)
**Alcohol (servings/d)**	0.9 (0.7)	0.0 (-1.6 to 1.2)	0.5 (0.4)	0.0 (-1.0 to 0.6)	0.3 (0.4)	0.0 (-0.4 to 0.6)
**Total fruit without juice (servings/d)**	1.5 (0.7)	0.1 (-1.5 to 1.8)	1.5 (0.5)	0.0 (-1.5 to 1.5)	1.2 (0.8)	0.0 (-1.0 to 1.1)
**Total vegetables (servings/d)**	2.9 (1.0)	0.2 (-2.2 to 3.2)	3.2 (0.8)	0.1 (-2.2 to 2.8)	3.1 (1.7)	0.0 (-2.2 to 2.4)
**Whole-fat dairy (servings/d)**	1.0 (0.6)	-0.1 (-1.9 to 1.1)	1.2 (0.5)	-0.1 (-1.8 to 1.0)	0.8 (0.7)	0.0 (-1.1 to 0.9)
**Low-fat dairy (servings/d)**	0.9 (0.6)	-0.1 (-1.5 to 1.5)	0.9 (0.4)	0.1 (-1.1 to 1.6)	1.1 (0.9)	0.0 (-1.1 to 1.3)
**Seafood (servings/d)**	0.4 (0.2)	0.0 (-0.5 to 0.4)	0.3 (0.1)	0.0 (-0.4 to 0.4)	0.3 (0.2)	0.0 (-0.3 to 0.3)
**Whole grains (servings/d)**	1.5 (0.8)	0.0 (-1.9 to 2.4)	0.8 (0.4)	0.1 (-1.3 to 1.8)	1.2 (1.0)	0.0 (-1.2 to 1.1)
**Refined grains (servings/d)**	1.2 (0.6)	0.0 (-1.9 to 1.8)	1.2 (0.5)	0.0 (-1.4 to 1.4)	1.3 (0.8)	-0.1 (-1.1 to 1.3)
**Nuts (servings/d)**	0.3 (0.3)	0.0 (-0.7 to 0.7)	0.1 (0.1)	0.0 (-0.4 to 0.4)	0.1 (0.1)	0.1 (-0.1 to 0.6)
**Sugar-sweetened beverages (servings/d)**	0.3 (0.4)	0.0 (-0.8 to 0.6)	0.2 (0.2)	0.0 (-0.5 to 0.5)	0.3 (0.6)	0.0 (-0.7 to 0.6)
**Juice (servings/d)**	0.8 (0.5)	0.0 (-1.2 to 1.2)	0.7 (0.4)	0.0 (-1.0 to 1.0)	0.6 (0.7)	-0.1 (-0.9 to 0.7)
**Sweets (servings/d)**	1.3 (0.8)	0.0 (-2.0 to 1.9)	1.2 (0.5)	0.0 (-1.4 to 1.8)	1.2 (0.9)	-0.1 (-1.2 to 1.1)
**Processed meats (servings/d)**	0.4 (0.3)	0.0 (-0.7 to 0.4)	0.3 (0.2)	0.0 (-0.5 to 0.4)	0.2 (0.2)	0.0 (-0.3 to 0.4)
**Trans fat (%)**	1.3 (0.3)	0.0 (-0.6 to 1.1)	1.7 (0.3)	-0.2 (-1.0 to 0.7)	1.6 (0.5)	-0.2 (-0.9 to 0.4)

**Fig 1:**
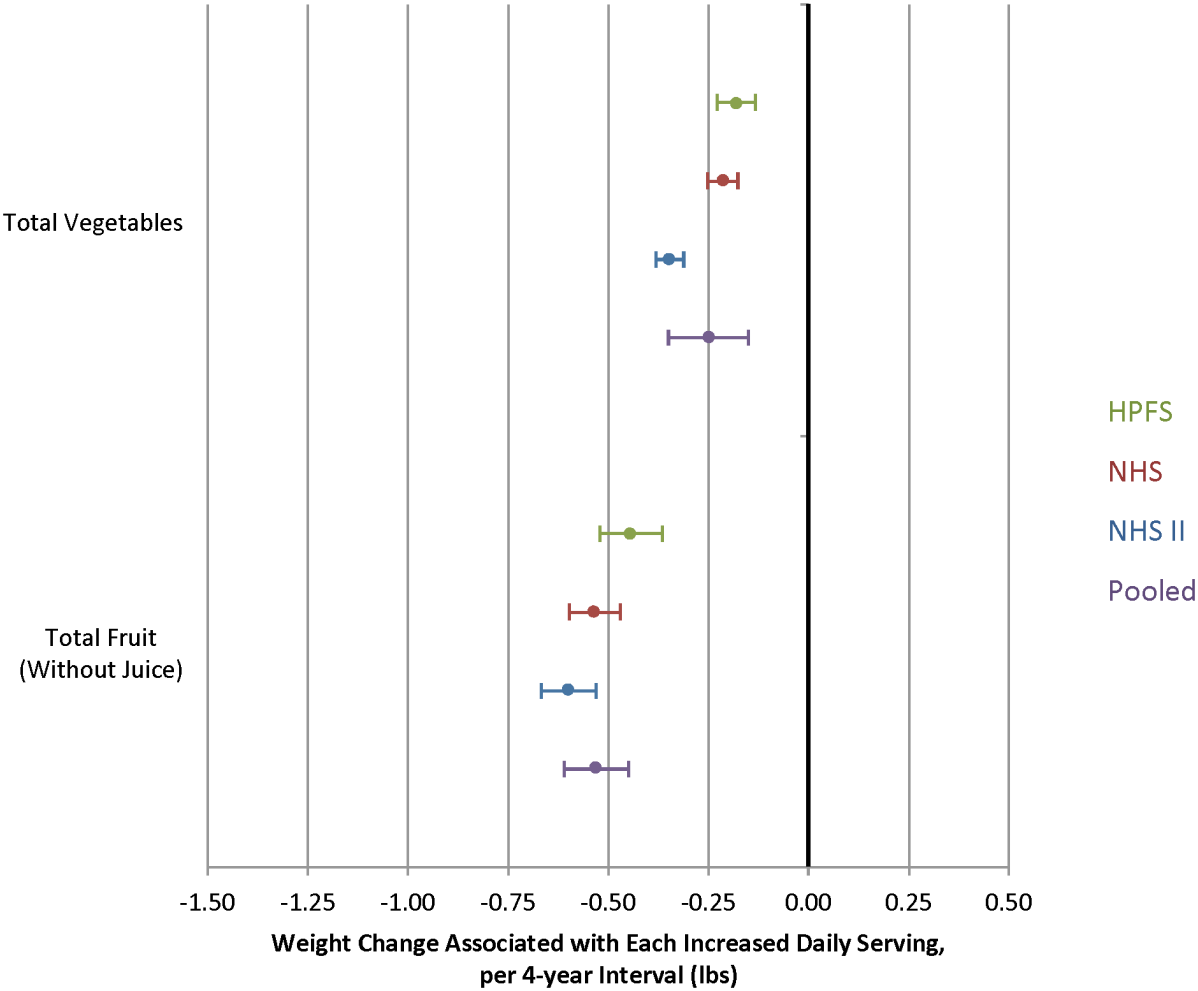
Relationships between changes in total vegetable and total fruit intake and weight change over 4 y in three cohorts. **Total vegetables**: string beans, broccoli, cabbage/coleslaw, cauliflower, Brussels sprouts, carrots (raw, cooked, or juice), corn, peas, lima beans, mixed vegetables or vegetable soup, beans, lentils, celery, squash, eggplant, zucchini, yams, sweet potatoes, baked/boiled/mashed potatoes, spinach, kale, mustard or chard greens, iceberg or head lettuce, romaine or leaf lettuce, peppers, tomatoes, onions, tofu and soy (soy burger, soybeans, miso, or other soy protein). **Total fruit (without juice)**: raisins, grapes, avocados, bananas, cantaloupe, watermelon, apples, pears, peaches (fresh or canned), apricots (fresh or canned), plums (fresh or canned), strawberries, blueberries, prunes, oranges, grapefruit (fresh or juice). Adjusted for baseline age and BMI and change in the following lifestyle variables: smoking status, physical activity, hours of sitting or watching TV, hours of sleep, fried potatoes, juice, whole grains, refined grains, fried foods, nuts, whole-fat dairy, low-fat dairy, sugar-sweetened beverages, sweets, processed meats, non-processed meats, *trans* fat, alcohol, and seafood.

An increase in both total fruit intake and total vegetable intake was inversely associated with weight change in all three cohorts (Fig 1). Pooled across all three cohorts, increased intake of vegetables was associated with a weight change of -0.25 lb per daily serving over 4y (95% CI, -0.35 to -0.14 lb), and fruits, -0.53 lb per daily serving (95% CI, -0.61 to -0.44 lb).

**Fig 2:**
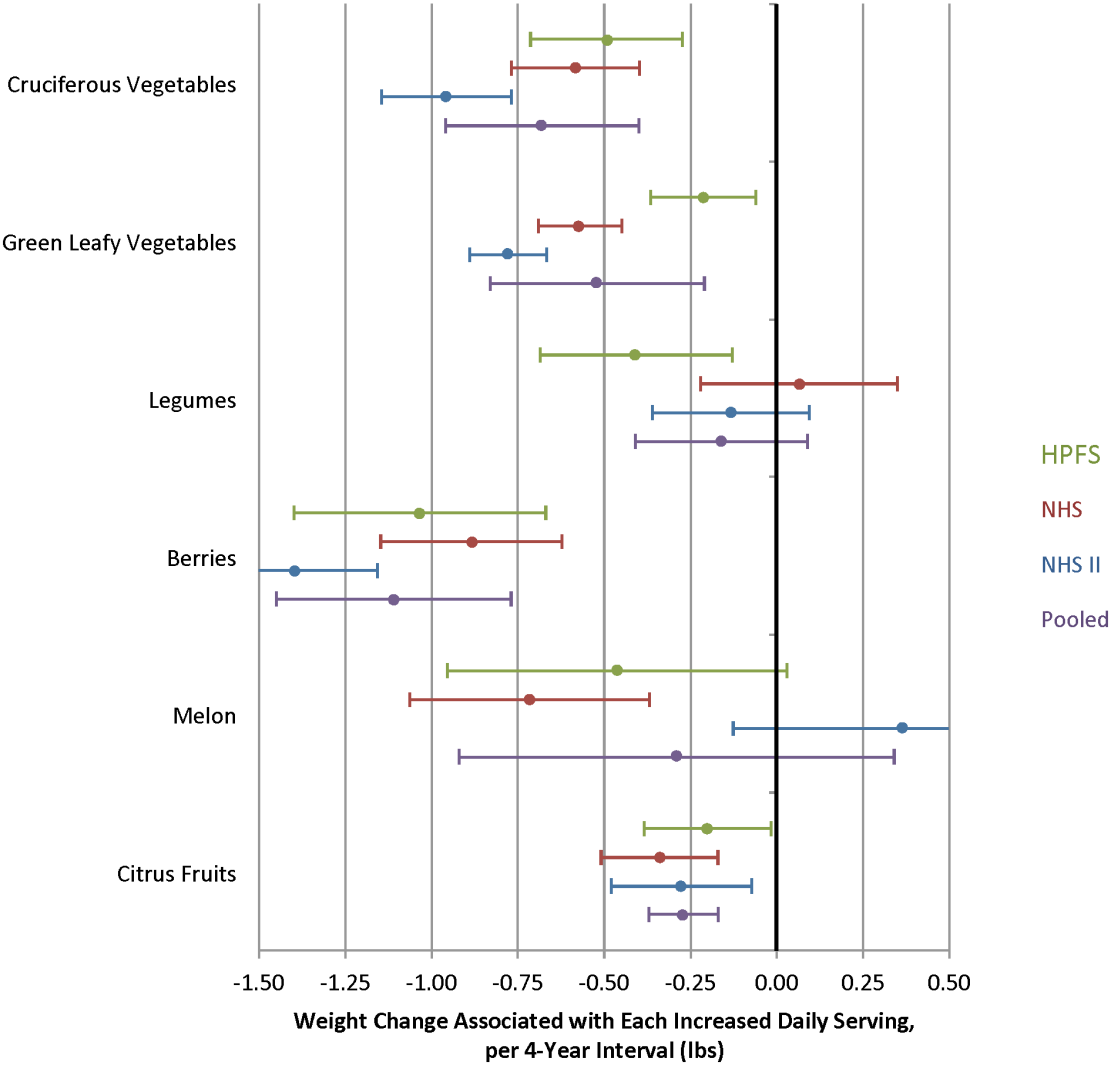
Relationships between changes in intake of classes of vegetables and fruits and weight change over 4 y in three cohorts. **Cruciferous vegetables**: broccoli, cauliflower, cabbage, Brussels sprouts. **Green leafy vegetables**: kale, mustard or chard greens, spinach, head or romaine lettuce. **Legumes**: peas, lima beans, beans, lentils, tofu/soy. **Berries**: blueberries, strawberries. **Melon**: cantaloupe, watermelon. **Citrus fruits**: oranges, grapefruit (fresh or juice). Adjusted for baseline age and BMI and change in the following lifestyle variables: smoking status, physical activity, hours of sitting or watching TV, hours of sleep, fried potatoes, juice, whole grains, refined grains, fried foods, nuts, whole-fat dairy, low-fat dairy, sugar-sweetened beverages, sweets, processed meats, non-processed meats, *trans* fat, alcohol, and seafood.

Evaluating specific subgroups of vegetables, increased intakes of cruciferous and green leafy vegetables was inversely associated with weight change: pooled change -0.68 lb per daily serving of cruciferous vegetables (95% CI, 0.96 to -0.40 lb) and -0.52 lb per daily serving of green leafy vegetables (95% CI, -0.83 to -0.22 lb) (Fig 2). Among subgroups of fruits, increased intakes of berries and citrus fruits were inversely associated with weight change: pooled change -1.11 lb (95% CI, -1.45 to -0.78 lb) for berries and -0.27 lb (95% CI, -0.37 to -0.17 lb) for citrus fruits.

**Fig 3:**
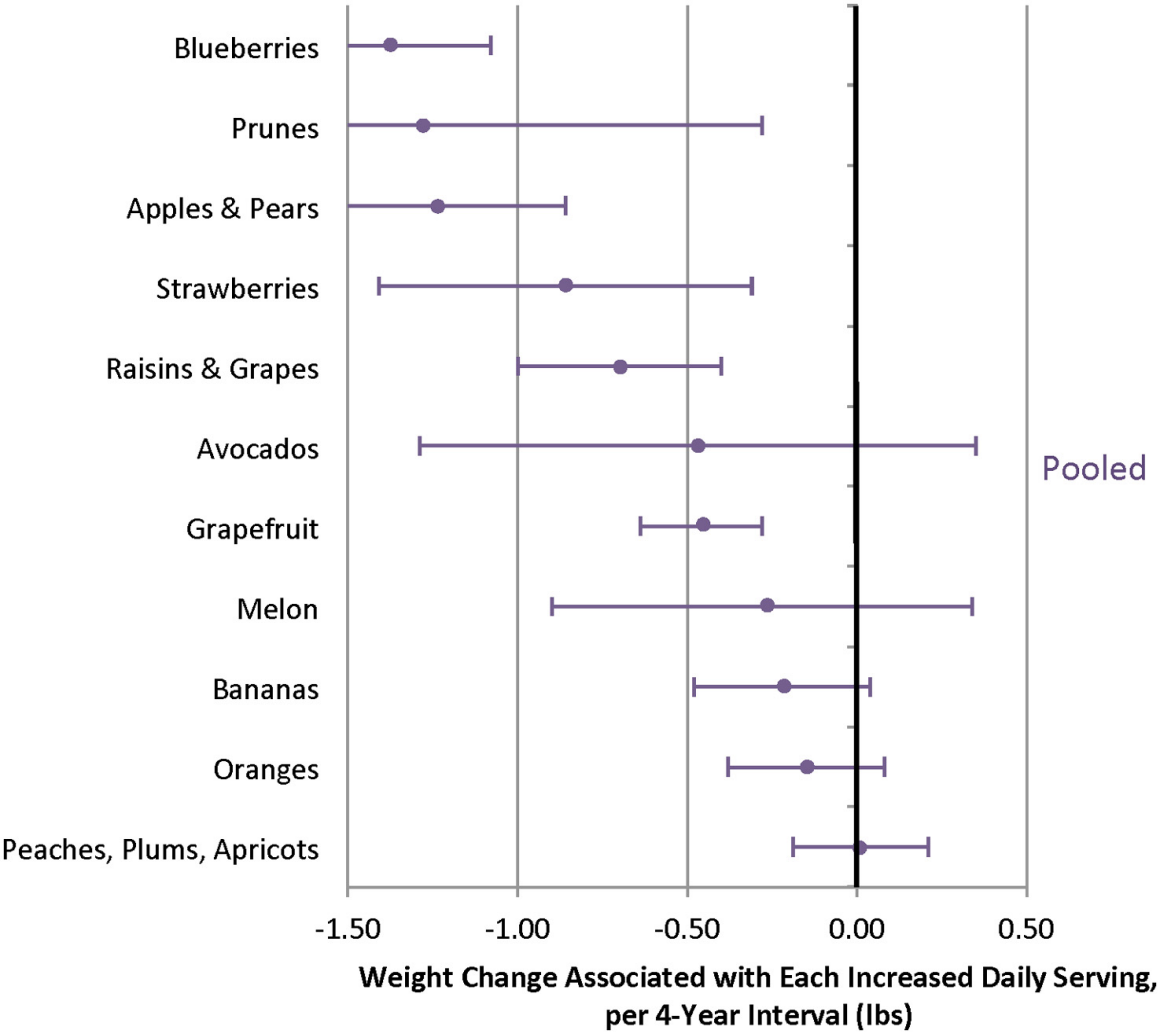
Relationships between changes in intake of specific fruits and weight change over 4 y in three cohorts. Adjusted for baseline age and BMI and change in the following lifestyle variables: smoking status, physical activity, hours of sitting or watching TV, hours of sleep, fried potatoes, juice, whole grains, refined grains, fried foods, nuts, whole-fat dairy, low-fat dairy, sugar-sweetened beverages, sweets, processed meats, non-processed meats, *trans* fat, alcohol, and seafood.

**Fig 4:**
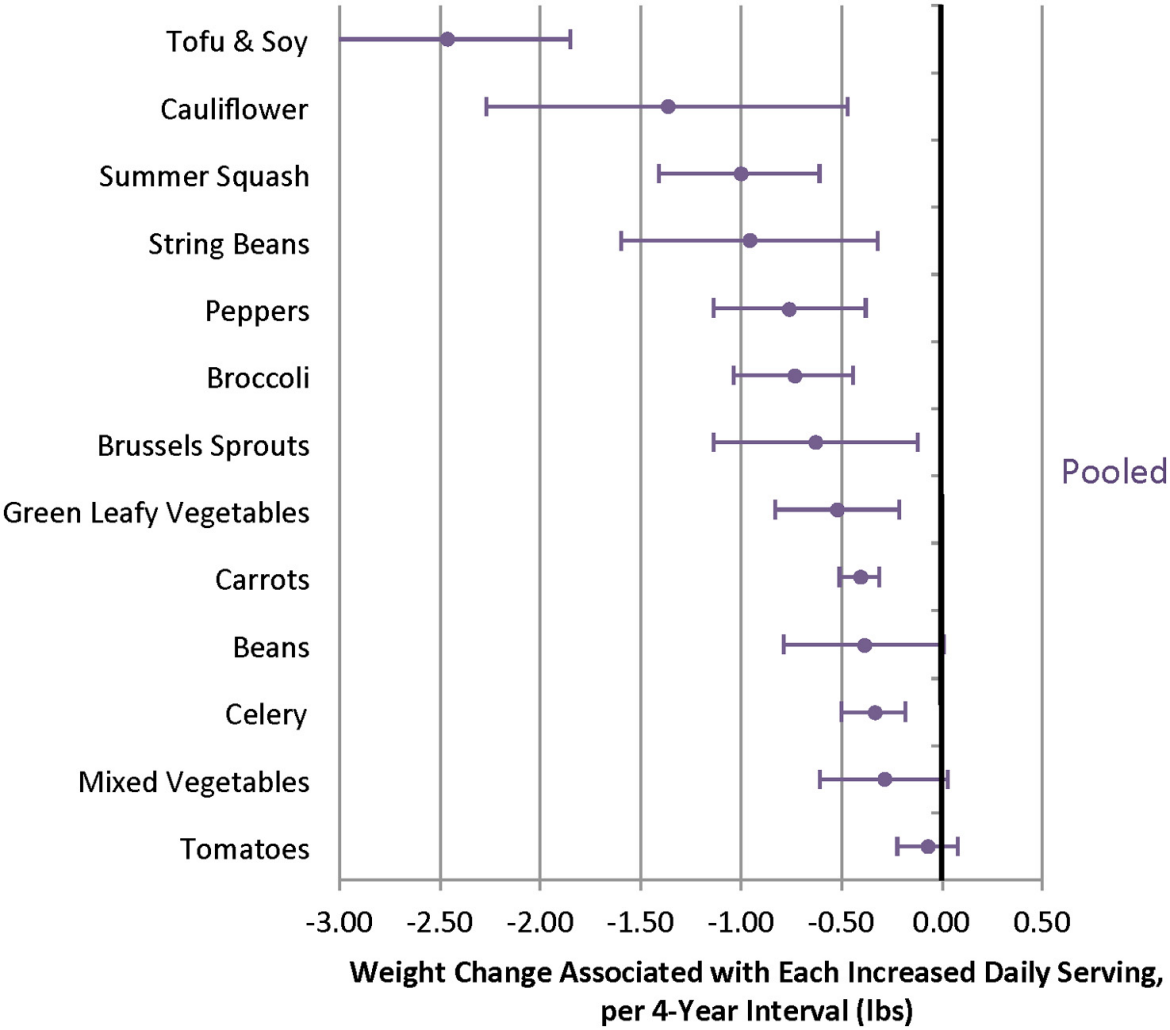
Relationships between changes in intake of specific vegetables and weight change over 4 y in three cohorts. Adjusted for baseline age and BMI and change in the following lifestyle variables: smoking status, physical activity, hours of sitting or watching TV, hours of sleep, fried potatoes, juice, whole grains, refined grains, fried foods, nuts, whole-fat dairy, low-fat dairy, sugar-sweetened beverages, sweets, processed meats, non-processed meats, *trans* fat, alcohol, and seafood.

**Fig 5:**
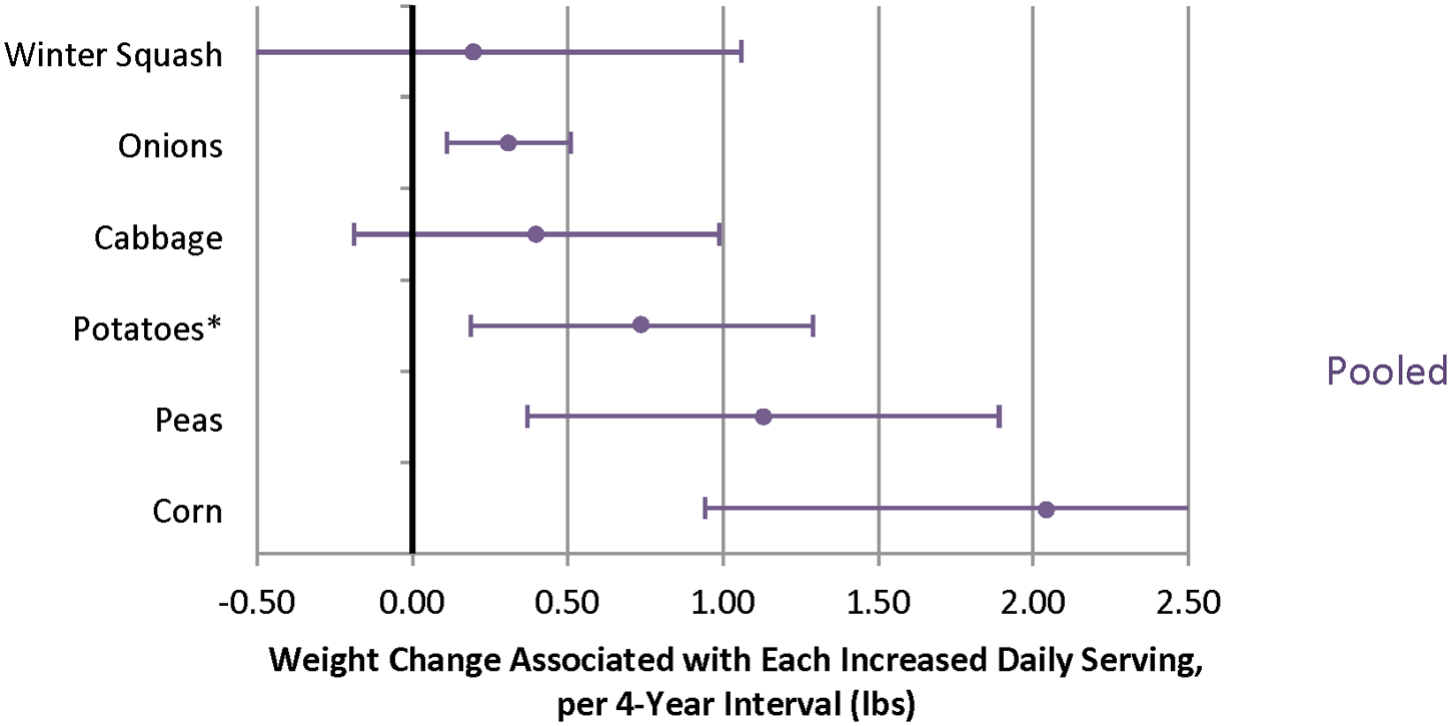
Relationships between changes in intake of specific vegetables and weight change over 4 y in three cohorts. *Includes baked/boiled/mashed white potatoes, sweet potatoes and yams; excludes french fries and potato chips. Adjusted for baseline age and BMI and change in the following lifestyle variables: smoking status, physical activity, hours of sitting or watching TV, hours of sleep, fried potatoes, juice, whole grains, refined grains, fried foods, nuts, whole-fat dairy, low-fat dairy, sugar-sweetened beverages, sweets, processed meats, non-processed meats, *trans* fat, alcohol, and seafood.

When different fruits were evaluated, increased intakes of several individual fruits were inversely associated with weight change over 4 y, including blueberries, prunes, apples/pears, strawberries, raisins/grapes, and grapefruit (Fig 3, S11 and S13 Tables). Increased intakes of many individual vegetables were also inversely associated with weight change, including tofu/soy (-2.47 lb; 95% CI -3.09 to -1.85 lb), peppers (-0.76 lb; 95% CI -1.14 to -0.39 lb), and carrots (-0.41 lb; 95% CI -0.51 to -0.42 lb) (Fig 4). Not all vegetables were inversely associated with weight change, however, most notably starchy vegetables. For example, additional daily servings of baked, boiled or mashed potatoes (0.74 lb; 95% CI 0.19 to 1.30 lb), peas (1.13 lb; 95% CI 0.37 to 1.89 lb), or corn (2.04 lb; 95% CI 0.94 to 3.15 lb) were each positively associated with weight change (Fig 5). Changes in intakes of specific fruits and vegetables were not highly correlated (S14–S16 Tables).

### Fiber Content and Weight Change

**Fig 6:**
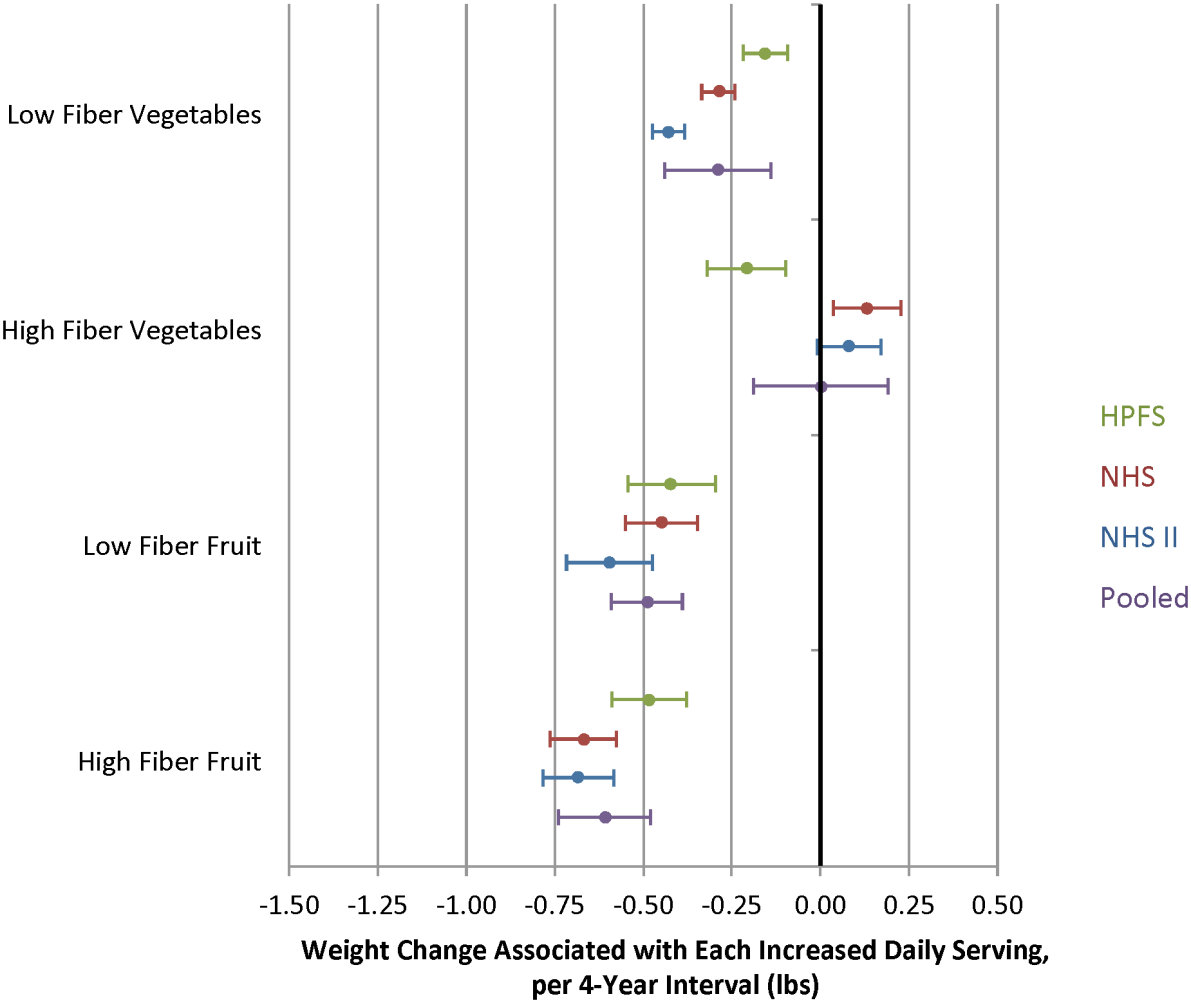
Relationships between changes in intake of fruits and vegetables classified as either low or high fiber and weight change over 4 y in three cohorts. Adjusted for baseline age and BMI and change in the following lifestyle variables: smoking status, physical activity, hours of sitting or watching TV, hours of sleep, fried potatoes, juice, whole grains, refined grains, fried foods, nuts, whole-fat dairy, low-fat dairy, sugar-sweetened beverages, sweets, processed meats, non-processed meats, *trans* fat, alcohol, and seafood.

**Fig 7:**
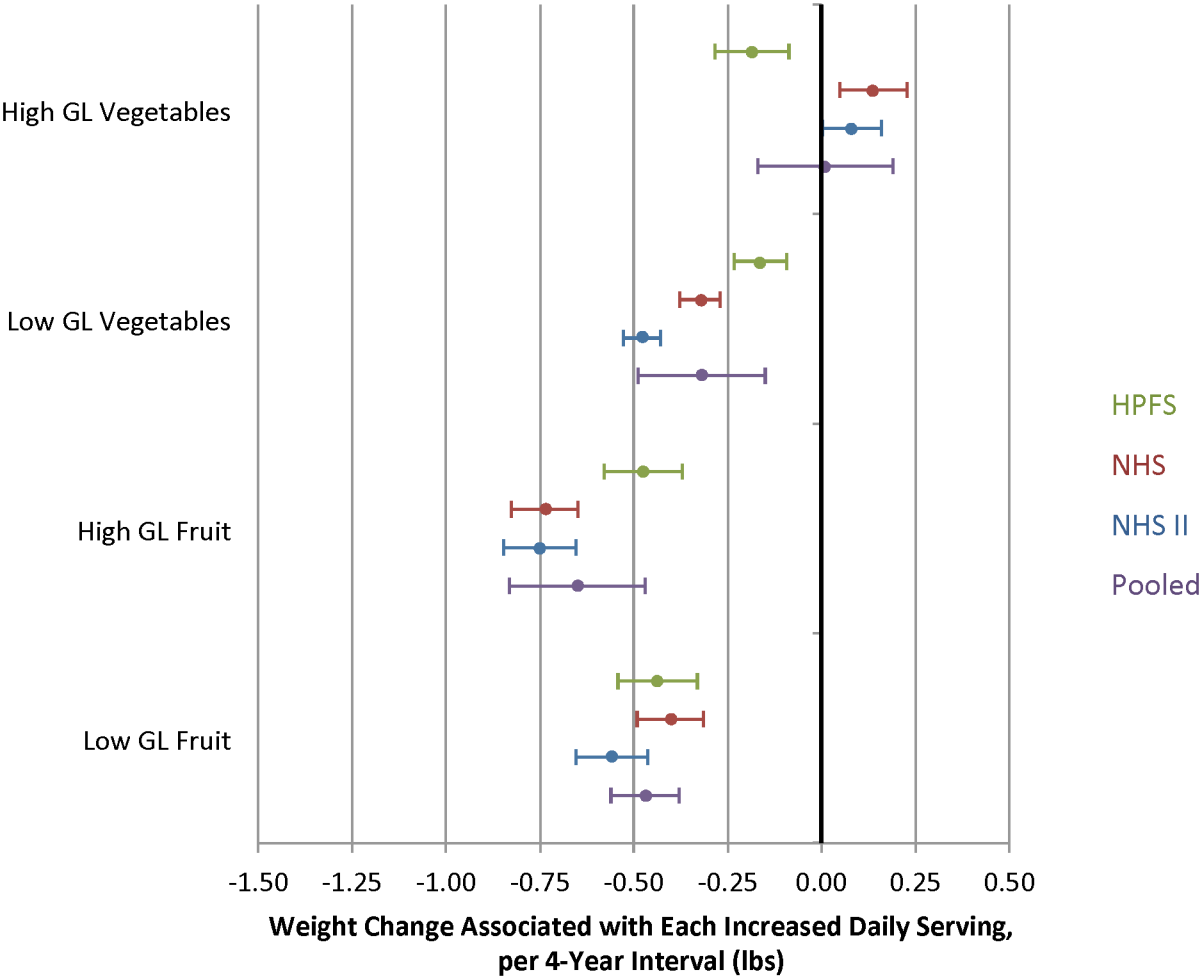
Relationships between changes in intake of fruits and vegetables classified as either high or low glycemic load (GL) and weight change over 4 y in three cohorts. Adjusted for baseline age and BMI and change in the following lifestyle variables: smoking status, physical activity, hours of sitting or watching TV, hours of sleep, fried potatoes, juice, whole grains, refined grains, fried foods, nuts, whole-fat dairy, low-fat dairy, sugar-sweetened beverages, sweets, processed meats, non-processed meats, *trans* fat, alcohol, and seafood.

The association between fruit intake and weight change was not modified by the fiber content (pooled p 0.16) or GL (pooled p 0.06) of the individual fruit. Thus, the benefits of greater fruit intake were seen regardless of the fiber content or GL (Figs 6 and 7, S2 and S3 Tables). Increased intake of lower-fiber vegetables was associated with negative weight change (-0.29 lb; 95% CI -0.44 to -0.14 lb) whereas increased intake of higher-fiber vegetables was not associated with weight change (0.00 lb; 95% CI -0.19 to 0.20 lb). However, when we excluded white potatoes (baked, boiled, or mashed) from the high-fiber subgroup, increased intake was associated with negative weight change (-0.19 lb; 95% CI -0.31 to -0.07 lb) (S8 Table).

### GL and Weight Change

When we categorized vegetables as either lower or higher GL (S4 and S5 Tables), lower-GL vegetables were inversely associated with weight change (Fig 7), a difference that was marginally statistically significant (pooled *p* = 0.05). An increase of one daily serving of a higher-GL vegetable was not associated with weight change (–0.01 lb; 95% CI -0.17 to 0.20 lb) whereas an increase of one daily serving of a lower-GL vegetable was associated with negative weight change (-0.32 lb; 95% CI -0.49 to -0.15 lb). Compared to vegetables that were both lower fiber and higher GL, we found greater negative weight change for higher-fiber, lower-GL vegetables (S1 Fig, pooled *p*-value < 0.0001).

Compared to lower-fiber, higher-GL fruits, weight change for higher-fiber, lower-GL fruits was similar: pooled change -0.40 lb per increased daily serving of higher-fiber, lower-GL fruits (95% CI -0.58 to -0.21 lb) versus -0.57 lb per increased daily serving of lower-fiber, higher-GL fruits (95% CI -0.80 to -0.35 lb). We found no evidence of effect modification by fiber content or GL of fruits.

### Sensitivity Analyses

Excluding individuals with missing diet, weight, or covariate information during follow-up and controlling for baseline levels of BMI and total fruit and vegetable intake did not appreciably change our results (S9 and S12 Tables). Additionally, adjusting for change in total energy intake to estimate the association between increased fruit and vegetable intake and weight change independent of changes in total energy produced similar results (Table 2). Increasing the relative proportion of total calories from fruit and vegetables in the diet was also inversely associated with weight change. Finally, using non-isocaloric substitution models, replacing 5% of calories from other foods with 5% of calories from fruits or vegetables was also associated with negative weight change.

**Table 2:** Energy sensitivity analyses: Weight change (lb) associated with increased consumption of fruits and vegetables over 4 y.

	Main analysis	Sensitivity analysis #1	Sensitivity analysis #2	Sensitivity analysis #3
	Increase of 1 serving per day	Increase of 1 serving per day, adjusted for change in total energy	Increase of 1 serving per day, energy-adjusted (residual method)	5% increase in energy
**Total fruits**				
HPFS	-0.44 (-0.52, -0.36)	-0.48 (-0.56 to -0.40)	-0.46 (-0.54 to -0.37)	-0.54 (-0.63 to -0.44)
NHS	-0.53 (-0.60, -0.47)	-0.53 (-0.60 to -0.47)	-0.53 (-0.60 to -0.46)	-1.96 (-2.25 to -1.68)
NHS II	-0.60 (-0.67, -0.53)	-0.61 (-0.68 to -0.54)	-0.67 (-0.74 to -0.59)	-0.99 (-1.07 to -0.91)
**Pooled**	**-0.53 (-0.61, -0.44)**	**-0.54 (-0.61 to -0.47)**	**-0.55 (-0.67 to -0.43)**	**-1.14 (-1.64 to -0.63)**
**Total vegetables**				
HPFS	-0.18 (-0.23, -0.13)	-0.20 (-0.25 to -0.15)	-0.20 (-0.25 to -0.15)	-0.61 (-0.73 to -0.49)
NHS	-0.21 (-0.25, -0.18)	-0.21 (-0.25 to -0.17)	-0.22 (-0.26 to -0.17)	-1.05 (-1.56 to -0.54)
NHS II	-0.35 (-0.38, -0.31)	-0.35 (-0.39 to -0.32)	-0.40 (-0.44 to -0.37)	-1.14 (-1.23 to -1.05)
**Pooled**	**-0.25 (-0.35, -0.14)**	**-0.25 (-0.36 to -0.15)**	**-0.27 (-0.41 to -0.14)**	**-0.92 (-1.35 to -0.49)**

Adjusted for baseline age and BMI and change in the following lifestyle variables: smoking status, physical activity, hours of sitting or watching TV, hours of sleep, fried potatoes, juice, whole grains, refined grains, fried foods, nuts, whole-fat dairy, low-fat dairy, sugar-sweetened beverages, sweets, processed meats, non-processed meats, *trans* fat, alcohol, and seafood.

When we stratified our analysis by weight at baseline (normal weight [BMI <25 kg/m^2^], overweight [BMI ≥25 and <30 kg/m^2^], and obese [BMI ≥30 kg/m^2^]) the negative weight change associated with greater intake of fruits and vegetables was stronger among overweight individuals compared to normal-weight individuals (S10 Table, *p*-values for interaction terms between total fruit and BMI 0.03 in HPFS, 0.06 in NHS, and 0.09 in NHS II; *p*-values for interaction terms between total vegetable intake and BMI 0.03 in all three cohorts). When we stratified our analysis by smoking status (current versus never or former), associations were similar for nonsmokers compared to current smokers (S10 Table).

## Discussion

In our 24-y prospective study with up to seven repeated dietary assessments, increased fruit and vegetable intake was inversely associated with weight change over time. The benefits were greater for fruits compared to vegetables and strongest for berries, apples/pears, tofu/soy, cauliflower, and cruciferous and green leafy vegetables. We found a stronger inverse association between increased intake of higher-fiber, lower-GL vegetables and weight change, consistent with experimental evidence suggesting an influence of these factors on satiety [8], glucose and insulin responses [21], fat storage [21], and energy expenditure [9].

We found that many vegetables were inversely associated with weight change, but starchy vegetables such as peas, potatoes, and corn had the opposite association in which increased intake was associated with weight gain. Although these vegetables have nutritional value (potassium, vitamin C, vitamin B_6_, iron, fiber, and protein), they have a higher GL (lower carbohydrate quality) that could explain their positive association with weight change.

Our models were not isocaloric because part of the benefit of fruits and vegetables may be from increased satiety with fewer calories; therefore the main results presented here are non-isocaloric substitutions in which individuals could have substituted, for example, one serving per day of apples (74 calories per serving) instead of one serving per day of orange juice (84 calories per serving). Alternatively, individuals could have added one serving of apples daily without changing other aspects of their diet. However, individuals will often replace one food item with another when they change their diet. Table 2 compares results from the main analyses that do not adjust for energy intake to results from various models that adjust for total energy, some of which estimate the effect of substitution.

In the first sensitivity analysis (Table 2), models are additionally adjusted for change in total energy intake. By controlling for change in total energy, this model estimates the association between increased fruit or vegetable intake and weight change independent of changes in total energy or, in other words, through mechanisms other than reduced calorie intake. These results are very similar to models that do not include energy intake; however, it is difficult to estimate total calorie intake precisely with FFQs. Therefore, these results should be interpreted with caution. This model allows total energy intake to change among individuals within each 4-y time interval; therefore, it is not isocaloric. This is not a substitution model because individuals could have replaced other foods with fruits and vegetables or they could have simply added more fruits and vegetables to their diet.

The second energy sensitivity analysis examines change in energy-adjusted fruit and vegetable intake. Energy adjustment using the residual method looks at the composition of the diet instead of absolute intake, in other words, fruit and vegetable intake relative to other individuals with the same total daily energy intake. These results are similar, suggesting that increasing the relative amount of fruits and vegetables in the diet is also negatively associated with weight change. Again, this is not a substitution model because individuals could have increased the proportion of fruits and vegetables in their diet by replacing other foods with fruits and vegetables or by increasing fruit and vegetable intake without changing other aspects of their diet. The third sensitivity analysis examines substitutions; however, it still allows total energy intake to change over time in individuals and therefore is not isocaloric. These results suggest that replacing 5% of calories from other foods with 5% of calories from fruits or vegetables is inversely associated with weight change.

Previous prospective studies of fruit and vegetable intake have mixed findings [22]. Among 373,803 participants in the European Prospective Investigation into Cancer and Nutrition cohort, there was no association between baseline fruit and vegetable intake and weight change over 5 y [23], but this study used a single baseline measure of diet that did not incorporate change over time. On the other hand, higher intake of fruits and vegetables was inversely associated with weight change over the following 6 y among 4,287 Australian women [24].

To the best of our knowledge, only three studies have used a change-on-change analysis [2,25,26] and one was a more general analysis of the population included in our study. Barone Gibbs et al. found a similar inverse association between increased fruit and vegetable intake (combined) and weight change over 42 mo among 481 women enrolled in a lifestyle intervention study [25]. Drapeau et al. found an inverse association between increased consumption of fruits but not vegetables and change in weight over 6 y among 248 individuals in the Quebec Family Study [27]. Previous clinical trials similarly have mixed findings: increased consumption of total fruits and vegetables over 3 mo was associated with weight loss among 103 overweight individuals with sleep-related eating disorders [28], but not over 6 mo among 690 healthy study participants [29], or over 2 mo in 50 healthy men and women [30].

Few studies have examined weight change in relation to specific fruits and vegetables; however, two trials examined interventions that included apples, pears, and grapefruit, all of which were beneficial in our population. Both trials found that increased intake resulted in weight loss—women randomized to eat apples or pears 3 times daily for 12 wk lost an average of 2.6 lb [31], while men and women randomized to eat three grapefruit halves daily for 6 wk lost an average of 1.3 lb [32]. Besides polyphenol content, fruits could be beneficial for maintaining or achieving a healthy weight if they are replacing less healthy desserts and snacks, which is often how they are consumed [33].

### Limitations

Our study has potential limitations. Although the study FFQ specified portion size, the assessment of diet using any method will have measurement error. However, this error is likely to be random and would tend to underestimate the association between intake of fruits and vegetables and weight change. Results could also be underestimated due to potential reverse causality if individuals who gain weight in the beginning of a 4-y time interval eat more fruits and vegetables later in the 4-y time interval in an effort to lose weight. Furthermore, the high correlation between measured and reported weight in our validation study could be overestimated if all individuals underreported weight by equal amounts.

Although we were able to adjust for changes in physical activity, we cannot rule out the possibility of residual confounding due to health consciousness if individuals who are eating healthier also make other healthier lifestyle changes not captured completely by our questionnaires. Although all participants were health professionals with graduate degrees, there remains a possibility of residual confounding due to unmeasured economic differences between participants within this strata of income and education. Furthermore, our study population consists mainly of white, educated adults. Therefore, our results may not be generalizable to all adults; however, it is unlikely that the biologic mechanisms underlying this association are different in other populations.

### Study Strengths

Strengths of our study include the repeated measurement of diet using a validated questionnaire over 24 y in over 100,000 adults. Due to the large sample size and long follow-up period, we had the unique opportunity to investigate not only change in total fruit and vegetable intake, but also intake of individual fruits and vegetables and fruits and vegetables classified by fiber content and GL. Looking at within-person change allowed us to control for stable personal characteristics such as gender and ethnicity. Furthermore, by restricting to educated participants with a higher socioeconomic status, and by consistently adjusting for major confounders across all three cohorts, we were able to reduce residual confounding by these factors and increase statistical power. Finally, we found consistent results across three cohorts that represent a wide range of ages and both genders.

In these three large cohorts, increasing consumption of all fruits and most vegetables was not associated with weight gain. Although the magnitude of weight change associated with each increased daily serving was modest, combining an increase of one-to-two servings of vegetables and one-to-two servings of fruits daily would be associated with substantial weight change, especially if projected to the population level. Furthermore, many individuals find it extremely difficult to lose weight, and therefore weight maintenance, as compared to weight gain, is an important goal. Simply maintaining weight from adulthood onward could have a substantial impact on population health.

We observed a robust inverse association between fruit and vegetable intake and long-term weight change in three large prospective cohorts of American adults. Unfortunately, most Americans have inadequate fruit and vegetable intake [34,35], and trends indicate that intake has remained relatively constant over time and may even be decreasing in some subgroups of the population [35–37]. Furthermore, although fruit juice and potato intakes have decreased over time, both still contribute substantially to total fruit and vegetable intake, and therefore public health recommendations and nutritional guidelines ought to emphasize individual or subgroups of specific fruits and vegetables that maximize the potential for weight maintenance and disease prevention [34]. In conclusion, our findings support benefits of increased fruit and vegetable consumption for preventing long-term weight gain and provide further food-specific guidance for the prevention of obesity, a primary risk factor for type 2 diabetes, cardiovascular diseases, cancers, and many other health conditions.

## Supporting Information

S1 Fig Weight change (lb) associated with an increase of one serving per day of fruits and vegetables categorized by fiber content and GL per serving. **Low-fiber, high-GL fruits**: melon, raisins, grapes. **Low-fiber, low-GL fruits**: strawberries, peaches, plums, apricots, grapefruit. **High-fiber, high-GL fruits**: prunes, apples, pears, bananas. **High-fiber, low-GL fruits**: avocados, blueberries, oranges. **Low-fiber, high-GL vegetables**: carrots, cabbage, coleslaw, sauerkraut. **Low-fiber, low-GL vegetables**: cauliflower, leafy greens, summer squash, tomatoes, peppers, celery, onions. **High-fiber, high-GL vegetables**: beans, lentils, tofu/soy, peas, lima beans, mixed vegetables, winter squash, potatoes, corn. **High-fiber, low-GL vegetables**: Brussels sprouts, broccoli, string beans. Adjusted for baseline age and BMI and change in the following lifestyle variables: smoking status, physical activity, hours of sitting or watching TV, hours of sleep, fried potatoes, juice, whole grains, refined grains, fried foods, nuts, whole-fat dairy, low-fat dairy, sugar-sweetened beverages, sweets, processed meats, non-processed meats, *trans* fat, alcohol, and seafood.
(TIFF)

S1 Table Frequency of physical activity, hours of watching TV, and hours of sleeping data collection. (DOCX)

S2 Table Fiber content of fruits included on the study FFQ. (DOCX)

S3 Table Fiber content of vegetables included on the study FFQ. (DOCX)

S4 Table Glycemic load (GL), glycemic index (GI), and calories per serving of fruits included on the study FFQ. (DOCX)

S5 Table Glycemic load (GL), glycemic index (GI), and calories per serving of vegetables included on the study FFQ. (DOCX)

S6 Table Definitions of fruits and vegetables. (DOCX)

S7 Table Food frequency questionnaire fruit and vegetable serving sizes. (DOCX)

S8 Table Weight change (lb) associated with an increase of one serving per day of fruits and vegetables classified as high or low fiber and GL, excluding potatoes, *n* = 133,468 men and women. (DOCX)

S9 Table Weight change (lb) associated with an increase of one serving per day of total fruits and total vegetables using a complete case analysis, additionally adjusting for baseline fruit and vegetable intake and weight, and using weight change in the future 4-y interval. (DOCX)

S10 Table Weight change (lb) associated with an increase of one serving per day of total fruits and total vegetables stratified by BMI and smoking status. (DOCX)

S11 Table Q-statistic for heterogeneity between the three cohorts. (DOCX)

S12 Table Modeling sensitivity analyses: weight change (lb) associated with increased consumption of fruits and vegetables over 4 y with and without dietary covariates, and with and without updated covariates. (DOCX)

S13 Table Cohort-specific associations for specific fruits and vegetables. (DOCX)

S14 Table Intercorrelations between changes in food intake 1986–1990: Results from the Health Professionals Follow-up Study. (DOCX)

S15 Table Intercorrelations between changes in food intake 1986–1990: Results from the Nurses' Health Study I. (DOCX)

S16 Table Intercorrelations between changes in food intake 1986–1990: Results from the Nurses' Health Study II. (DOCX)

S17 Table Pearson correlation coefficients (r) between mean consumption of fruits and vegetables estimated by dietary record (DR) and food frequency questionnaire (FFQ) among men in the Health Professionals Follow-up Study. (DOCX)

S18 Table Exclusions (sequential) at baseline. (DOCX)

S19 Table Baseline (mean, SD) fruit and vegetable intake (servings/day) of men and women in three prospective cohorts. (DOCX)

S1 Text STROBE statement. (DOC)

S2 Text A priori project proposal. (DOC)

## Abbreviations

BMI: body mass index
FFQ: food frequency questionnaire
GI: glycemic index
GL: glycemic load
HPFS: Health Professionals Follow-up Study
NHS: Nurses’ Health Study
NHS II: Nurses’ Health Study II
USDA: United States Department of Agriculture
