# Correction: Changes in Intake of Fruits and Vegetables and Weight Change in United States Men and Women Followed for Up to 24 Years: Analysis from Three Prospective Cohort Studies

**DOI:** 10.1371/journal.pmed.1001956

**Published:** 2016-01-20

**Authors:** Monica L. Bertoia, Kenneth J. Mukamal, Leah E. Cahill, Tao Hou, David S. Ludwig, Dariush Mozaffarian, Walter C. Willett, Frank B. Hu, Eric B. Rimm

There are errors in the Funding section. The correct funding information is as follows: This study was supported by grants P01 CA87969, R01 CA49449, R01 HL034594, R01 HL088521, UM1 CA176726, R01 CA67262, UM1 CA167552, R01 HL35464, and K24DK082730 from the National Institutes of Health and the project ‘Diet and prevention of ischemic heart disease: a translational approach’ (DIPI, www.dipi.dk), which is supported by the Danish Council for Strategic Research (today, Innovation Fund Denmark) (Contract 0603-00488B). The funders had no role in study design, data collection and analysis, decision to publish, or preparation of the manuscript.
