# Supplementary figures and images for: Changes in Intake of Fruits and Vegetables and Weight Change in United States Men and Women Followed for Up to 24 Years: Analysis from Three Prospective Cohort Studies
Source: PLoS Med. 2015 Sep 22;12(9):e1001878. doi: 10.1371/journal.pmed.1001878 (PMC4578962; doi:10.1371/journal.pmed.1001878)

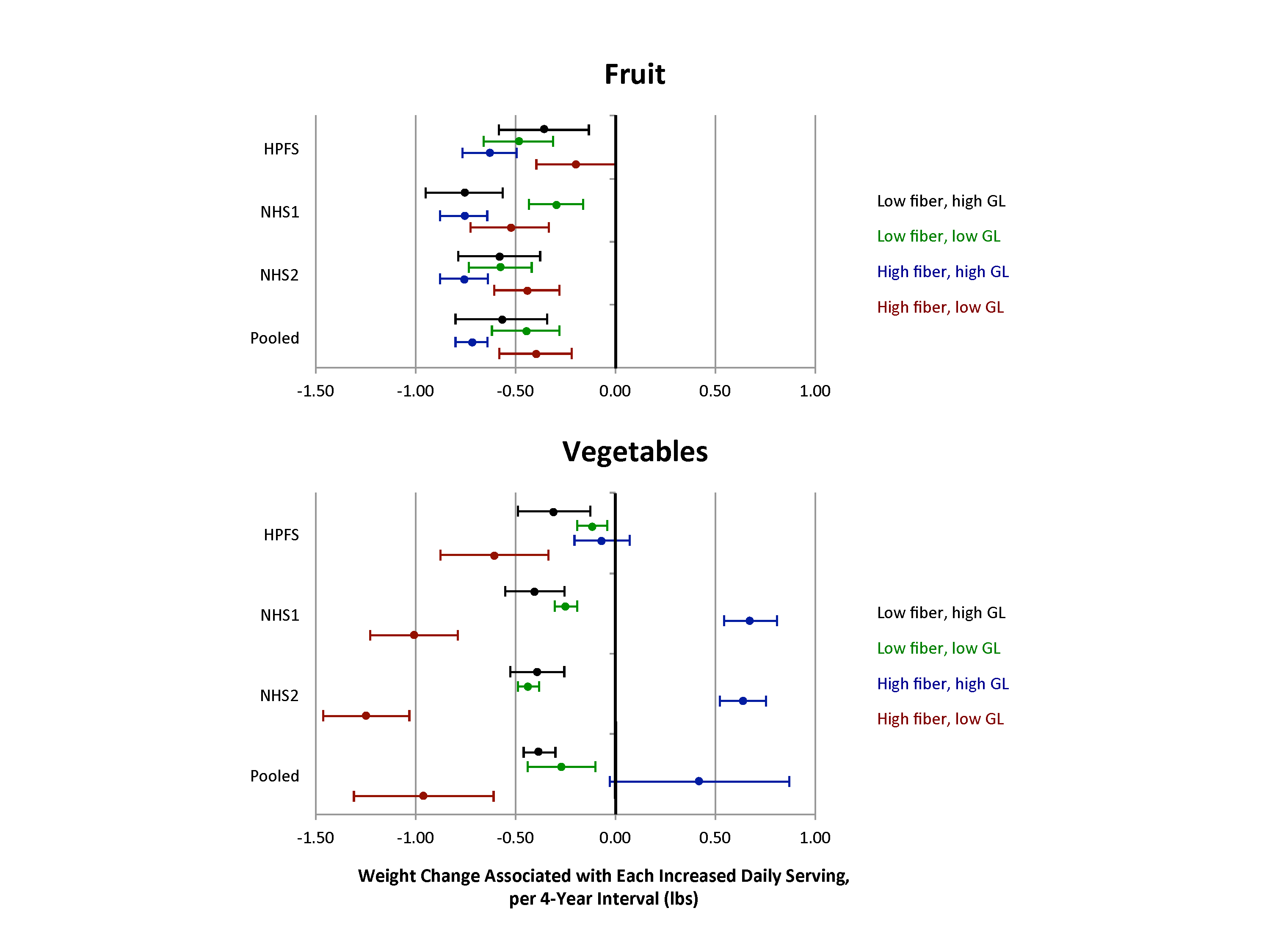

Supplement: S1 Fig — Low-fiber, high-GL fruits: melon, raisins, grapes. Low-fiber, low-GL fruits: strawberries, peaches, plums, apricots, grapefruit. High-fiber, high-GL fruits: prunes, apples, pears, bananas. High-fiber, low-GL fruits: avocados, blueberries, oranges. Low-fiber, high-GL vegetables: carrots, cabbage, coleslaw, sauerkraut. Low-fiber, low-GL vegetables: cauliflower, leafy greens, summer squash, tomatoes, peppers, celery, onions. High-fiber, high-GL vegetables: beans, lentils, tofu/soy, peas, lima beans, mixed vegetables, winter squash, potatoes, corn. High-fiber, low-GL vegetables: Brussels sprouts, broccoli, string beans. Adjusted for baseline age and BMI and change in the following lifestyle variables: smoking status, physical activity, hours of sitting or watching TV, hours of sleep, fried potatoes, juice, whole grains, refined grains, fried foods, nuts, whole-fat dairy, low-fat dairy, sugar-sweetened beverages, sweets, processed meats, non-processed meats, trans fat, alcohol, and seafood. (TIFF) [file pmed.1001878.s001.tiff]
