# Supplementary material for: Changes in Intake of Fruits and Vegetables and Weight Change in United States Men and Women Followed for Up to 24 Years: Analysis from Three Prospective Cohort Studies
Source: PLoS Med. 2015 Sep 22;12(9):e1001878. doi: 10.1371/journal.pmed.1001878 (PMC4578962; doi:10.1371/journal.pmed.1001878)
Supplement: S1 Table — (DOCX) [file pmed.1001878.s002.docx]

**Supplemental Table 1. Frequency of physical activity, hours of watching TV, and hours of sleeping data collection.**

| **Covariate** | **HPFS** | **NHS** | **NHS II** |
| --- | --- | --- | --- |
| Physical activity | Data collected biennially. | Data collected biennially. | Data collected in 1991, 1997, 2001, and 2005.  Data from 1991 was used to impute values for 1993 and 1995, data from 1997 for 1999, data from 2001 for 2003, and data from 2005 for 2007. |
| Hours of watching TV | Data collected in 1998 and every 2 years thereafter.  Four-year change included as a covariate in each model assuming no change between 1986 and 1998. | Data collected in 1992, 2004, and 2008.  Baseline levels rather than change variables were included in each model due to the infrequent timing of collection: data from 1992 was used to impute values for 1986, 1990, 1994, 1998, and 2002. Data from 2004 was used to impute values for 2006. | Data collected in 1991, 1997, 2001, and 2005.  Baseline levels rather than change variables were included in each model due to the infrequent timing of collection: data from 1991 was used to impute values for 1995, data from 1997 for 1999, and data from 2001 for 2003. |
| Hours of sleep | Data collected in 1987 and in 2000.  Baseline levels rather than change variables were included in each model due to the infrequent timing of collection: data from 1987 was used to impute values for 1986, 1990, 1994, and 1998. Data from 2000 for 2002 and 2006. | Data collected in 1986, 2000, 2002, and 2008.  Baseline levels rather than change variables were included in each model due to the infrequent timing of collection: carrying forward data for 1990, 1994, 1998, and 2006. | Data collected in 2001.  Data from 2001 used as a covariate in all models. |
