# Supplementary material for: Changes in Intake of Fruits and Vegetables and Weight Change in United States Men and Women Followed for Up to 24 Years: Analysis from Three Prospective Cohort Studies
Source: PLoS Med. 2015 Sep 22;12(9):e1001878. doi: 10.1371/journal.pmed.1001878 (PMC4578962; doi:10.1371/journal.pmed.1001878)
Supplement: S2 Table — (DOCX) [file pmed.1001878.s003.docx]

| **Supplemental Table 2. Fiber content of fruits included on the study FFQ** | | | | |  |  |
| --- | --- | --- | --- | --- | --- | --- |
| **Fruits** | |  | **g Fiber/serving** | **g Carb/serving** | **Carb:fiber ratio** | **Cal/serving** |
|  | High fiber | |  |  |  |  |
|  |  | Avocados | 6.7 | 8.5 | 1.3 | 161 |
|  |  | Prunes | 3.8 | 33.2 | 8.7 | 125 |
|  |  | Apples, pears | 3.6 | 20.0 | 5.6 | 75 |
|  |  | Oranges | 3.1 | 15.5 | 5.0 | 62 |
|  |  | Bananas | 3.0 | 26.0 | 8.7 | 101 |
|  |  | Blueberries | 1.8 | 10.6 | 5.9 | 42 |
|  |  | **Average** | **3.7** | **19.0** | **5.9** | **94** |
|  | Low fiber | |  |  |  |  |
|  |  | Strawberries | 1.5 | 5.8 | 3.9 | 24 |
|  |  | Peaches, plums, apricots | 1.4 | 13.1 | 9.4 | 51 |
|  |  | Grapefruit, grapefruit juice | 1.3 | 9.7 | 7.5 | 38 |
|  |  | Cantaloupe, watermelon | 1.2 | 11.0 | 9.2 | 46 |
|  |  | Raisins, grapes | 1.0 | 20.8 | 20.8 | 79 |
|  |  | **Average** | **1.3** | **12.1** | **10.1** | **48** |
