# Supplementary material for: Changes in Intake of Fruits and Vegetables and Weight Change in United States Men and Women Followed for Up to 24 Years: Analysis from Three Prospective Cohort Studies
Source: PLoS Med. 2015 Sep 22;12(9):e1001878. doi: 10.1371/journal.pmed.1001878 (PMC4578962; doi:10.1371/journal.pmed.1001878)
Supplement: S3 Table — (DOCX) [file pmed.1001878.s004.docx]

| **Supplemental Table 3. Fiber content of vegetables included on the study FFQ** | | | | | |  |
| --- | --- | --- | --- | --- | --- | --- |
| **Vegetables** | | | **g Fiber/serving** | **g Carb/serving** | **Carb:fiber ratio** | **Cal/serving** |
|  | High fiber | |  |  |  |  |
|  |  | Beans, lentils | 8.4 | 28.6 | 3.4 | 159 |
|  |  | Tofu, soybeans, soy burger, miso, other soy protein | 4.7 | 9.0 | 1.9 | 123 |
|  |  | Peas, lima beans | 4.4 | 13.5 | 3.1 | 73 |
|  |  | Mixed, stir-fry vegetables | 4.0 | 11.9 | 3.0 | 59 |
|  |  | Baked/mashed potatoes, yams, sweet potatoes | 3.5 | 32.9 | 9.4 | 144 |
|  |  | Brussels sprouts | 3.2 | 6.5 | 2.0 | 33 |
|  |  | Winter squash | 2.9 | 9.1 | 3.1 | 38 |
|  |  | Broccoli | 2.6 | 5.6 | 2.2 | 27 |
|  |  | String beans | 2.0 | 4.4 | 2.2 | 19 |
|  |  | Corn | 2.0 | 15.8 | 7.9 | 66 |
|  |  | **Average** | **3.8** | **13.7** | **3.8** | **74** |
|  | Low fiber | |  |  |  |  |
|  |  | Carrots | 1.7 | 4.9 | 2.9 | 21 |
|  |  | Cabbage, coleslaw, sauerkraut | 1.6 | 8.6 | 5.4 | 85 |
|  |  | Cauliflower | 1.4 | 2.5 | 1.8 | 14 |
|  |  | Spinach, kale, mustard greens, iceberg/romaine lettuce | 1.4 | 2.6 | 1.9 | 14 |
|  |  | Eggplant, zucchini | 1.3 | 3.5 | 2.7 | 14 |
|  |  | Tomatoes | 1.1 | 3.5 | 3.2 | 16 |
|  |  | Peppers | 0.3 | 0.7 | 2.3 | 3 |
|  |  | Celery | 0.3 | 9.1 | 30.3 | 3 |
|  |  | Onions | 0.2 | 1.3 | 6.5 | 6 |
|  |  | **Average** | **1.0** | **4.1** | **6.3** | **20** |
