# Supplementary material for: Changes in Intake of Fruits and Vegetables and Weight Change in United States Men and Women Followed for Up to 24 Years: Analysis from Three Prospective Cohort Studies
Source: PLoS Med. 2015 Sep 22;12(9):e1001878. doi: 10.1371/journal.pmed.1001878 (PMC4578962; doi:10.1371/journal.pmed.1001878)
Supplement: S4 Table — (DOCX) [file pmed.1001878.s005.docx]

| **Supplemental Table 4. Glycemic load (GL), glycemic index (GI), and calories per serving of fruits included on the study FFQ** | | | | | |
| --- | --- | --- | --- | --- | --- |
| **Fruits** |  |  | **GL** | **GI** | **Cal/serving** |
|  | Low GL | |  |  |  |
|  |  | Avocados | 0.5 | 5.9 | 161 |
|  |  | Strawberries | 2.3 | 30.0 | 24 |
|  |  | Grapefruit, grapefruit juice | 2.4 | 30.2 | 38 |
|  |  | Blueberries | 5.6 | 38.7 | 42 |
|  |  | Peaches, plums, apricots | 5.7 | 50.5 | 51 |
|  |  | Oranges | 6.2 | 52.4 | 62 |
|  |  | **Average** | **3.8** | **34.6** | **63** |
|  | High GL | |  |  |  |
|  |  | Cantaloupe, watermelon | 7.6 | 93.8 | 46 |
|  |  | Apples, pears | 7.9 | 55.9 | 75 |
|  |  | Raisins, grapes | 13.0 | 42.2 | 79 |
|  |  | Prunes | 13.9 | 26.1 | 125 |
|  |  | Bananas | 16.1 | 70.7 | 101 |
|  |  | **Average** | **11.7** | **57.7** | **85** |
