# Supplementary material for: Changes in Intake of Fruits and Vegetables and Weight Change in United States Men and Women Followed for Up to 24 Years: Analysis from Three Prospective Cohort Studies
Source: PLoS Med. 2015 Sep 22;12(9):e1001878. doi: 10.1371/journal.pmed.1001878 (PMC4578962; doi:10.1371/journal.pmed.1001878)
Supplement: S5 Table — (DOCX) [file pmed.1001878.s006.docx]

| **Supplemental Table 5. Glycemic load (GL), glycemic index (GI), and calories per serving of vegetables included on the study FFQ** | | | | | |
| --- | --- | --- | --- | --- | --- |
| **Vegetables** | |  | **GL** | **GI** | **Cal/serving** |
|  | Low GL | |  |  |  |
|  |  | Peppers | 0.1 | 1.3 | 3 |
|  |  | Celery | 0.1 | 1.8 | 3 |
|  |  | Cauliflower | 0.2 | 5.6 | 14 |
|  |  | Spinach, kale, mustard greens, iceberg/romaine lettuce | 0.2 | 6.3 | 14 |
|  |  | String beans | 0.4 | 6.2 | 19 |
|  |  | Eggplant, zucchini | 0.4 | 8.2 | 14 |
|  |  | Onions | 0.4 | 4.5 | 6 |
|  |  | Tomatoes | 0.4 | 8.3 | 16 |
|  |  | Broccoli | 0.5 | 7.1 | 27 |
|  |  | Brussels sprouts | 0.6 | 7.1 | 33 |
|  |  | **Average** | **0.3** | **5.6** | **15** |
|  | High GL | |  |  |  |
|  |  | Carrots | 1.8 | 21.6 | 21 |
|  |  | Cabbage, coleslaw, sauerkraut | 2.1 | 18.3 | 85 |
|  |  | Winter squash | 3.7 | 41.3 | 38 |
|  |  | Mixed, stir-fry vegetables | 4.4 | 33.7 | 59 |
|  |  | Tofu, soybeans, soy burger, miso, other soy protein | 4.8 | 47.6 | 123 |
|  |  | Peas, lima beans | 6.0 | 37.0 | 73 |
|  |  | Beans, lentils | 9.0 | 41.4 | 159 |
|  |  | Corn | 9.7 | 50.0 | 66 |
|  |  | Baked/mashed potatoes, yams, sweet potatoes | 22.5 | 104.3 | 144 |
|  |  | **Average** | **7.1** | **43.9** | **85** |
