# Supplementary material for: Changes in Intake of Fruits and Vegetables and Weight Change in United States Men and Women Followed for Up to 24 Years: Analysis from Three Prospective Cohort Studies
Source: PLoS Med. 2015 Sep 22;12(9):e1001878. doi: 10.1371/journal.pmed.1001878 (PMC4578962; doi:10.1371/journal.pmed.1001878)
Supplement: S6 Table — (DOCX) [file pmed.1001878.s007.docx]

| **Supplemental Table 6. Definitions of fruits and vegetables.** | | |
| --- | --- | --- |
| Vegetables | |  |
|  | Total vegetables | String beans, broccoli, cabbage/coleslaw, cauliflower, Brussels sprouts, carrots (raw, cooked, or juice), corn, peas, lima beans, mixed vegetables or vegetable soup, beans, lentils, celery, squash, eggplant, zucchini, yams, sweet potatoes, baked/boiled/mashed potatoes, spinach, kale, mustard or chard greens, iceberg or head lettuce, romaine or leaf lettuce, peppers, tomatoes, onions, tofu and soy (soy burger, soybeans, miso, or other soy protein) |
|  | Legumes | Peas, lima beans, beans, lentils, tofu or soy |
|  | Cruciferous vegetables | Broccoli, cauliflower, cabbage, Brussels sprouts |
|  | Green leafy vegetables | Kale, mustard or chard greens, spinach, lettuce (head or romaine) |
|  | Potatoes | Baked, boiled or mashed potatoes, yams or sweet potatoes |
|  |  |  |
| Fruit | |  |
|  | Total fruit | Raisins, grapes, avocados, bananas, cantaloupe, watermelon, apples, pears, peaches (fresh or canned), apricots (fresh or canned), plums (fresh or canned), strawberries, blueberries, prunes, oranges, grapefruit (fresh or juice) |
|  | Melon | Cantaloupe, watermelon |
|  | Citrus fruits | Oranges, grapefruit (fresh or juice) |
|  | Berries | Strawberries, blueberries |
