# Supplementary material for: Changes in Intake of Fruits and Vegetables and Weight Change in United States Men and Women Followed for Up to 24 Years: Analysis from Three Prospective Cohort Studies
Source: PLoS Med. 2015 Sep 22;12(9):e1001878. doi: 10.1371/journal.pmed.1001878 (PMC4578962; doi:10.1371/journal.pmed.1001878)
Supplement: S7 Table — (DOCX) [file pmed.1001878.s008.docx]

| **Supplemental Table 7. Food frequency questionnaire fruit and vegetable serving sizes.** | | |
| --- | --- | --- |
| Fruits |  |  |
|  | Raisins | 1 oz or small pack |
|  | Grapes | 1/2 cup |
|  | Avocado | 1/2 fruit or 1/2 cup |
|  | Banana | 1 |
|  | Cantaloupe | 1/4 melon |
|  | Watermelon | 1 slice |
|  | Apples | 1 |
|  | Pears | 1 |
|  | Peaches, apricots or plums | 1 fresh, or 1/2 cup canned |
|  | Strawberries | 1/2 cup fresh, frozen or canned |
|  | Blueberries | 1/2 cup fresh, frozen or canned |
|  | Prunes | 6 dried or 1/4 cup canned |
|  | Oranges | 1 |
|  | Grapefruit | 1/2 |
|  | Grapefruit juice | Small glass |
|  |  |  |
| Vegetables | |  |
|  | String beans | 1/2 cup |
|  | Broccoli | 1/2 cup |
|  | Raw cabbage or coleslaw | 1/2 cup |
|  | Cooked cabbage or sauerkraut | 1/2 cup |
|  | Cauliflower | 1/2 cup |
|  | Brussels sprouts | 1/2 cup |
|  | Raw carrots | 1/2 carrot or 2-4 sticks |
|  | Cooked carrots | 1/2 cup |
|  | Carrot juice | 2-3 oz |
|  | Corn | 1 ear or 1/2 cup frozen or canned |
|  | Peas or lima beans | 1/2 cup fresh, frozen, canned |
|  | Mixed or stir-fry vegetables | 1/2 cup |
|  | Vegetable soup | 1 cup |
|  | Beans or lentils | 1/2 cup baked or dried |
|  | Celery | 2-3 sticks |
|  | Dark yellow/orange (winter) squash | 1/2 cup |
|  | Eggplant, zucchini, or other summer squash | 1/2 cup |
|  | Potatoes | 1 baked or boiled or 1 cup mashed |
|  | Yams or sweet potatoes | 1/2 cup |
|  | Cooked spinach | 1/2 cup |
|  | Raw spinach | 1 cup |
|  | Kale, mustard greens or chard | 1/2 cup |
|  | Iceberg or head lettuce | 1 serving |
|  | Romaine or leaf lettuce | 1 serving |
|  | Green, yellow or red peppers | 3 slices or 1/4 pepper |
|  | Tomatoes | 2 slices |
|  | Tofu or soybeans | 3-4 oz |
|  | Fresh onion | 1 slice |
|  | Cooked onion | 1/2 cup |
