# Supplementary material for: Changes in Intake of Fruits and Vegetables and Weight Change in United States Men and Women Followed for Up to 24 Years: Analysis from Three Prospective Cohort Studies
Source: PLoS Med. 2015 Sep 22;12(9):e1001878. doi: 10.1371/journal.pmed.1001878 (PMC4578962; doi:10.1371/journal.pmed.1001878)
Supplement: S8 Table — (DOCX) [file pmed.1001878.s009.docx]

| **Supplemental Table 8. Weight change (lbs) associated with an increase of one serving per day of fruits and vegetables classified as high or low fiber and GL, excluding potatoes, n = 133,468 men and women.** | | |
| --- | --- | --- |
|  | **Main Analysis** | **Excluding Potatoes*** |
| **High fiber fruit** | -0.61 (-0.74 to -0.49) | -0.62 (-0.74 to -0.49) |
| **Low fiber fruit** | -0.49 (-0.59 to -0.38) | -0.48 (-0.58 to -0.38) |
| **High fiber vegetables** | 0.00 (-0.19 to 0.20) | -0.19 (-0.31 to -0.07) |
| **Low fiber vegetables** | -0.29 (-0.44 to -0.14) | -0.28 (-0.43 to -0.14) |
|  |  |  |
| **Low GL fruit** | -0.47 (-0.56 to -0.37) | -0.45 (-0.55 to -0.36) |
| **High GL fruit** | -0.65 (-0.83 to -0.48) | -0.65 (-0.81 to -0.48) |
| **Low GL vegetables** | -0.32 (-0.49 to -0.15) | -0.30 (-0.46 to -0.14) |
| **High GL vegetables** | 0.01 (-0.17 to 0.20) | -0.10 (-0.24 to 0.05) |
| * Baked, boiled, or mashed white potatoes, yams, and sweet potatoes. | | |
| Adjusted for baseline age and BMI and change in the following lifestyle variables: smoking status, physical activity, hours of sitting or watching TV, hours of sleep, fried potatoes, juice, whole grains, refined grains, fried foods, nuts, whole-fat dairy, low-fat dairy, sugar sweetened beverages, sweets, processed meats, non-processed meats, *trans* fat, alcohol, and seafood. | | |
