# Supplementary material for: Changes in Intake of Fruits and Vegetables and Weight Change in United States Men and Women Followed for Up to 24 Years: Analysis from Three Prospective Cohort Studies
Source: PLoS Med. 2015 Sep 22;12(9):e1001878. doi: 10.1371/journal.pmed.1001878 (PMC4578962; doi:10.1371/journal.pmed.1001878)
Supplement: S9 Table — (DOCX) [file pmed.1001878.s010.docx]

| **Supplemental Table 9. Weight change (lbs) associated with an increase of one serving per day of total fruits and total vegetables using a complete case analysis, additionally adjusting for baseline fruit and vegetable intake and weight, and using weight change in the future 4-year interval.** | | | | | |
| --- | --- | --- | --- | --- | --- |
|  |  | Main Analysis:  missing diet and weight data carried forward one cycle | Complete Case:  individuals with missing diet or weight data excluded | Additionally adjusted for baseline total fruit intake, total vegetable intake, and weight | Weight change in future 4-year interval |
| **Total n** | | | | | |
|  | HPFS | 19,316 | 18,930 | 19,316 | 18,541 |
|  | NHS | 40,415 | 39,775 | 40,415 | 40,110 |
|  | NHS II | 73,737 | 65,649 | 73,737 | 71,540 |
|  | **Pooled** | **133,468** | **124,354** | **133,468** | **130,191** |
|  |  |  |  |  |  |
| **Total Fruit** | | | | | |
|  | HPFS | -0.44 (-0.52, -0.36) | -0.42 (-0.50, -0.34) | -0.52 (-0.60, -0.44) | -0.06 (-0.14, 0.02) |
|  | NHS | -0.53 (-0.60, -0.47) | -0.54 (-0.61, -0.47) | -0.59 (-0.66, -0.52) | 0.05 (-0.01, 0.12) |
|  | NHS II | -0.60 (-0.67, -0.53) | -0.74 (-0.82, -0.67) | -0.74 (-0.81, -0.67) | 0.11 (0.03, 0.19) |
|  | **Pooled** | **-0.53 (-0.61, -0.44)** | **-0.58 (-0.90, -0.27)** | **-0.62 (-0.74, -0.49)** | **0.03 (-0.05, 0.12)** |
|  |  |  |  |  |  |
| **Total Vegetables** | | | | | |
|  | HPFS | -0.18 (-0.23, -0.13) | -0.18 (-0.23, -0.13) | -0.17 (-0.22, -0.12) | 0.05 (0.00, 0.10) |
|  | NHS | -0.21 (-0.25, -0.18) | -0.25 (-0.29, -0.21) | -0.20 (-0.24, -0.16) | 0.11 (0.07, 0.15) |
|  | NHS II | -0.35 (-0.38, -0.31) | -0.47 (-0.51, -0.44) | -0.33 (-0.37, -0.29) | 0.04 (0.00, 0.08) |
|  | **Pooled** | **-0.25 (-0.35, -0.14)** | **-0.33 (-0.62, -0.04)** | **-0.23 (-0.34, -0.13)** | **0.07 (0.03, 0.11)** |
| Adjusted for baseline age and BMI and change in the following lifestyle variables: smoking status, physical activity, hours of sitting or watching TV, hours of sleep, fried potatoes, juice, whole grains, refined grains, fried foods, nuts, whole-fat dairy, low-fat dairy, sugar sweetened beverages, sweets, processed meats, non-processed meats, *trans* fat, alcohol, and seafood. | | | | | |
