# Supplementary material for: Changes in Intake of Fruits and Vegetables and Weight Change in United States Men and Women Followed for Up to 24 Years: Analysis from Three Prospective Cohort Studies
Source: PLoS Med. 2015 Sep 22;12(9):e1001878. doi: 10.1371/journal.pmed.1001878 (PMC4578962; doi:10.1371/journal.pmed.1001878)
Supplement: S10 Table — (DOCX) [file pmed.1001878.s011.docx]

| **Supplemental Table 10 Weight change (lbs) associated with an increase of one serving per day of total fruits and total vegetables stratified by BMI and smoking status.** | | | | | | | |
| --- | --- | --- | --- | --- | --- | --- | --- |
|  |  | **Main Analysis:**  (no BMI restriction) | **Normal Weight:**  BMI < 25 kg/m^2^ at baseline | **Overweight:**  BMI ≥25 kg/m^2^ and < 30 kg/m^2^ at baseline | **Obese:** BMI ≥ 30 kg/m^2^  at baseline | **Nonsmokers:**  not a current smoker at the beginning and end of each 4-year time interval | **Smokers:**  current smoker at the beginning and end of each 4-year time interval |
| **Total n** | | | | | | | |
|  | HPFS | 19,316 | 9,559 | 8,422 | 1,319 | 16,642 | 1,210 |
|  | NHS | 40,415 | 24,393 | 11,015 | 5,007 | 31,620 | 6,264 |
|  | NHS II | 73,737 | 49,534 | 14,844 | 9,406 | 63,240 | 6,875 |
|  | **Pooled** | **133,468** | **83,486** | **34,281** | **15,732** | **111,502** | **14,349** |
|  |  |  |  |  |  |  |  |
| **Total Fruit** | | | | | | | |
|  | HPFS | -0.44 (-0.52, -0.36) | -0.31 (-0.39, -0.22) | -0.54 (-0.67, -0.41) | 0.02 (-1.24, 1.29) | -0.46 (-0.54, -0.38) | -0.33 (-0.81, 0.15) |
|  | NHS | -0.53 (-0.60, -0.47) | -0.34 (-0.40, -0.27) | -0.71 (-0.85, -0.57) | -0.99 (-1.29, -0.69) | -0.55 (-0.62, -0.48) | -0.53 (-0.73, -0.34) |
|  | NHS II | -0.60 (-0.67, -0.53) | -0.35 (-0.42, -0.29) | -0.84 (-1.02, -0.65) | -1.38 (-1.71, -1.06) | -0.59 (-0.66, -0.52) | -0.57 (-0.87, -0.27) |
|  | **Pooled** | **-0.53 (-0.61, -0.44)** | **-0.34 (-0.37, -0.30)** | **-0.69 (-0.86, -0.52)** | **0.79 (-1.52, -0.07)** | **-0.53 (-0.61, -0.46)** | **-0.52 (-0.66, -0.38)** |
|  |  |  |  |  |  |  |  |
| **Total Vegetables** | | | | | | | |
|  | HPFS | -0.18 (-0.23, -0.13) | -0.11 (-0.16, -0.06) | -0.22 (-0.30, -0.14) | -0.97 (-1.69, -0.25) | -0.20 (-0.25, -0.15) | -0.10 (-0.32, 0.13) |
|  | NHS | -0.21 (-0.25, -0.18) | -0.05 (-0.08, -0.01) | -0.38 (-0.46, -0.29) | -0.58 (-0.75, -0.42) | -0.25 (-0.29, -0.21) | -0.05 (-0.16, 0.06) |
|  | NHS II | -0.35 (-0.38, -0.31) | -0.20 (-0.23, -0.17) | -0.59 (-0.68, -0.51) | -0.59 (-0.75, -0.44) | -0.37 (-0.41, -0.33) | -0.16 (-0.28, -0.04) |
|  | **Pooled** | **-0.25 (-0.35, -0.14)** | **-0.12 (-0.22, -0.02)** | **-0.40 (-0.60, -0.19)** | **-0.70 (-0.92, -0.49)** | **-0.27 (-0.38, -0.17)** | **-0.10 (-0.17, -0.03)** |
| Adjusted for baseline age and BMI and change in the following lifestyle variables: smoking status (BMI stratified analyses only), physical activity, hours of sitting or watching TV, hours of sleep, fried potatoes, juice, whole grains, refined grains, fried foods, nuts, whole-fat dairy, low-fat dairy, sugar sweetened beverages, sweets, processed meats, non-processed meats, *trans* fat, alcohol, and seafood. | | | | | | | |
