# Supplementary material for: Changes in Intake of Fruits and Vegetables and Weight Change in United States Men and Women Followed for Up to 24 Years: Analysis from Three Prospective Cohort Studies
Source: PLoS Med. 2015 Sep 22;12(9):e1001878. doi: 10.1371/journal.pmed.1001878 (PMC4578962; doi:10.1371/journal.pmed.1001878)
Supplement: S11 Table — (DOCX) [file pmed.1001878.s012.docx]

| **Supplemental Table 11. Q-statistic for heterogeneity between the three cohorts.** | | | |
| --- | --- | --- | --- |
| **Fruit/Vegetable** | **Q-statistic** | **Fruit/Vegetable** | **Q-statistic** |
| Total fruits | 0.01 | Peaches, plums, apricots | 0.32 |
| Total vegetables | <0.0001 | Raisins &grapes | 0.03 |
|  |  | Avocados | 0.95 |
| Low GL fruits | 0.04 | Bananas | 0.01 |
| High GL fruits | <0.0001 | Melon | 0.001 |
| Low GL vegetables | <0.0001 | Apples & pears | <0.0001 |
| High Gl vegetables | <0.0001 | Strawberries | 0.01 |
|  |  | Blueberries | 0.87 |
| High fiber fruits | 0.01 | Prunes | <0.0001 |
| Low fiber fruits | 0.08 | Oranges | 0.06 |
| High fiber vegetables | <0.0001 | Grapefruit | 0.15 |
| Low fiber vegetables | <0.0001 | String beans | 0.0006 |
|  |  | Broccoli | 0.11 |
| Melon | 0.001 | Cabbage | 0.02 |
| Citrus fruits | 0.5 | Cauliflower | <0.0001 |
| Berries | 0.01 | Brussels sprouts | 0.76 |
| Legumes | 0.05 | Carrots | 0.32 |
| Cruciferous vegetables | 0.0007 | Corn | <0.0001 |
| Green leafy vegetables | <0.0001 | Peas | 0.0004 |
|  |  | Mixed vegetables | 0.11 |
|  |  | Beans | 0.07 |
|  |  | Celery | 0.47 |
|  |  | Winter squash | 0.07 |
|  |  | Summer squash | 0.23 |
|  |  | Green leafy vegetables | <0.0001 |
|  |  | Peppers | 0.05 |
|  |  | Tomatoes | 0.15 |
|  |  | Tofu/soy | 0.08 |
|  |  | Onions | 0.02 |
|  |  | Potatoes | <0.0001 |
