# Supplementary material for: Changes in Intake of Fruits and Vegetables and Weight Change in United States Men and Women Followed for Up to 24 Years: Analysis from Three Prospective Cohort Studies
Source: PLoS Med. 2015 Sep 22;12(9):e1001878. doi: 10.1371/journal.pmed.1001878 (PMC4578962; doi:10.1371/journal.pmed.1001878)
Supplement: S12 Table — (DOCX) [file pmed.1001878.s013.docx]

| **Supplemental Table 12. Modeling sensitivity analyses: weight change (lbs) associated with increased consumption of fruits and vegetables over four years with and without dietary covariates & with and without updated covariates.** | | | | |  |
| --- | --- | --- | --- | --- | --- |
|  |  | **Main analysis** | **No dietary covariates** | **No updated covariates** | |
| **Total fruits** | |  |  |  | |
|  | HPFS | -0.44 (-0.52, -0.36) | -0.53 (-0.60 to -0.45) | -0.46 (-0.54 to -0.37) | |
|  | NHS | -0.53 (-0.60, -0.47) | -0.64 (-0.70 to -0.57) | -0.50 (-0.62 to -0.38) | |
|  | NHS II | -0.60 (-0.67, -0.53) | -0.67 (-0.74 to -0.60) | -0.75 (-0.84 to -0.66) | |
|  | **Pooled** | **-0.53 (-0.61, -0.44)** | **-0.61 (-0.70 to -0.53)** | **-0.57 (-0.76 to -0.38)** | |
|  |  |  |  |  | |
| **Total vegetables** | | |  |  | |
|  | HPFS | -0.18 (-0.23, -0.13) | -0.15 (-0.20 to -0.11) | -0.19 (-0.25 to -0.14) | |
|  | NHS | -0.21 (-0.25, -0.18) | -0.20 (-0.23 to -0.16) | -0.21 (-0.28 to -0.14) | |
|  | NHS II | -0.35 (-0.38, -0.31) | -0.34 (-0.37 to -0.31) | -0.47 (-0.52 to -0.42) | |
|  | **Pooled** | **-0.25 (-0.35, -0.14)** | **-0.23 (-0.34 to -0.12)** | **-0.29 (-0.49 to -0.10)** | |
| Adjusted for baseline age and BMI and change in the following lifestyle variables: smoking status, physical activity, hours of sitting or watching TV, hours of sleep, and the following aspects of diet (analyses with dietary covariates only): fried potatoes, juice, whole grains, refined grains, fried foods, nuts, whole-fat dairy, low-fat dairy, sugar sweetened beverages, sweets, processed meats, non-processed meats, *trans* fat, alcohol, and seafood. | | | | |  |
