# Supplementary material for: Changes in Intake of Fruits and Vegetables and Weight Change in United States Men and Women Followed for Up to 24 Years: Analysis from Three Prospective Cohort Studies
Source: PLoS Med. 2015 Sep 22;12(9):e1001878. doi: 10.1371/journal.pmed.1001878 (PMC4578962; doi:10.1371/journal.pmed.1001878)
Supplement: S13 Table — (DOCX) [file pmed.1001878.s014.docx]

| **Supplemental Table 13. Cohort-specific associations for specific fruits and vegetables.** | | | | |  |
| --- | --- | --- | --- | --- | --- |
| **Fruits** | | **HPFS** | **NHS** | **NHS II** | **Pooled** |
|  | Blueberries | -1.35 (-1.81, -0.89) | -1.33 (-1.85, -0.82) | 1.53 (-2.15, -0.91) | -1.38 (-1.68, -1.09) |
|  | Prunes | -2.46 (-3.20, -1.72) | -0.73 (-1.10, -0.36) | -0.73 (-1.45, -0.02) | -1.28 (-2.28, -0.28) |
|  | Apples & pears | -1.45 (-1.65, -1.25) | -1.43 (-1.62, -1.23) | -0.85 (-1.06, -0.64) | -1.24 (-1.62, -0.86) |
|  | Strawberries | -1.35 (-1.72, -0.98) | -0.58 (-0.97, -0.19) | -0.60 (-1.21, 0.02) | -0.86 (-1.41, -0.31) |
|  | Raisins & grapes | -0.92 (-1.19, -0.64) | -0.79 (-1.04, -0.54) | -0.39 (-0.67, -0.11) | -0.70 (-1.00, -0.40) |
|  | Avocados | -0.49 (-1.93, 0.94) | -0.24 (-1.88, 1.40) | -0.57 (-1.74, 0.61) | -0.47 (-1.29, 0.34) |
|  | Grapefruit | -0.56 (-0.83, -0.28) | -0.29 (-0.50, -0.09) | -0.56 (-0.81, -0.32) | -0.46 (-0.64, -0.27) |
|  | Melon | 0.35 (-0.14, 0.84) | -0.70 (1.05, -0.35) | -0.45 (-0.94, 0.05) | -0.28 (-0.90, 0.34) |
|  | Bananas | -0.04 (-0.25, 0.17) | -0.13 (-0.33, 0.07) | -0.48 (-0.69, -0.27) | -0.22 (-0.48, 0.04) |
|  | Oranges | -0.07 (-0.35, 0.22) | -0.37 (-0.60, -0.13) | -0.01 (-0.25, 0.23) | -0.15 (-0.38, 0.08) |
|  | Peaches, plums, apricots | 0.14 (-0.20, 0.49) | 0.06 (-0.24, 0.35) | -0.25 (-0.67, 0.16) | 0.01 (-0.19, 0.22) |
| **Vegetables** | |  |  |  |  |
|  | Tofu & soy | -2.68 (-3.20, -2.17) | -2.92 (-3.80, -2.04) | -1.81 (-2.58, -1.03) | -2.47 (-3.09, -1.85) |
|  | Cauliflower | -2.14 (-2.66, -1.62) | 1.47 (-1.96, -0.97) | -0.47 (-1.07, 0.14) | -1.37 (-2.27, -0.47) |
|  | Summer squash | -0.88 (-1.43, -0.32) | -1.37 (-1.91, -0.82) | -0.68 (-1.40, 0.03) | -1.01 (-1.41, -0.61) |
|  | String beans | -1.29 (-1.68, -0.90) | -1.32 (-1.73, -0.91) | -0.18 (-0.72, 0.36) | -0.96 (-1.60, -0.32) |
|  | Peppers | -1.05 (-1.35, -0.75) | -0.48 (-0.87, -0.08) | -0.70 (-1.25, -0.14) | -0.76 (-1.14, -0.39) |
|  | Broccoli | -0.95 (-1.28, -0.61) | -0.47 (-0.81, -0.12) | -0.81 (-1.24, -0.37) | -0.74 (-1.04, -0.44) |
|  | Brussels sprouts | -0.79 (-1.82, 0.24) | -0.75 (-1.70, 0.20) | -0.36 (-1.34, 0.61) | -0.63 (-1.14, -0.12) |
|  | Green leafy vegetables | -0.78 (-0.89, -0.67) | -0.57 (-0.69, -0.45) | -0.21 (-0.36, -0.05) | -0.52 (-0.83, -0.21) |
|  | Carrots | -0.46 (-0.60, -0.32) | -0.43 (-0.60, -0.27) | -0.29 (-0.49, -0.09) | -0.41 (-0.51, -0.32) |
|  | Beans | -0.30 (-0.67, 0.08) | -0.07 (-0.54, 0.41) | -0.80 (-1.26, -0.34) | -0.39 (-0.79, 0.01) |
|  | Celery | -0.35 (-0.65, -0.04) | -0.43 (-0.67, -0.18) | -0.18 (-0.52, 0.16) | -0.34 (-0.50, -0.19) |
|  | Mixed vegetables | -0.56 (-0.90, -0.22) | -0.01 (-0.44, 0.41) | -0.25 (-0.67, 0.18) | -0.29 (-0.61, 0.03) |
|  | Tomatoes | -0.21 (-0.41, -0.01) | -0.02 (-0.21, 0.18) | 0.04 (-0.18, 0.26) | -0.07 (-0.22, 0.09) |
|  | Winter squash | -0.50 (-1.50, 0.49) | 0.95 (0.12, 1.78) | 0.06 (-0.93, 1.04) | 0.20 (-0.66, 1.07) |
|  | Onions | 0.47 (0.31, 0.63) | 0.35 (0.18, 0.52) | 0.08 (-0.16, 0.33) | 0.31 (0.11, 0.52) |
|  | Cabbage | 0.94 (0.33, 1.55) | 0.42 (-0.11, 0.94) | -0.14 (-0.71, 0.43) | 0.40 (-0.19, 0.99) |
|  | Potatoes* | 1.16 (0.91, 1.42) | 0.89 (0.66, 1.13) | 0.16 (-0.11, 0.44) | 0.74 (0.19, 1.30) |
|  | Peas | 1.78 (1.33, 2.23) | 1.16 (0.69, 1.62) | 0.42 (-0.10, 0.94) | 1.13 (0.37, 1.89) |
|  | Corn | 2.83 (2.45, 3.21) | 2.38 (1.92, 2.84) | 0.88 (0.35, 1.42) | 2.04 (0.94, 3.15) |
|  | *Includes baked/boiled/mashed white potatoes, sweet potatoes, and yams; excludes french fries and potato chips. | | | | |
| Adjusted for baseline age and BMI and change in the following lifestyle variables: smoking status, physical activity, hours of sitting or watching TV, hours of sleep, fried potatoes, juice, whole grains, refined grains, fried foods, nuts, whole-fat dairy, low-fat dairy, sugar sweetened beverages, sweets, processed meats, non-processed meats, *trans* fat, alcohol, and seafood. | | | | | |
