# Supplementary material for: Changes in Intake of Fruits and Vegetables and Weight Change in United States Men and Women Followed for Up to 24 Years: Analysis from Three Prospective Cohort Studies
Source: PLoS Med. 2015 Sep 22;12(9):e1001878. doi: 10.1371/journal.pmed.1001878 (PMC4578962; doi:10.1371/journal.pmed.1001878)
Supplement: S16 Table — (DOCX) [file pmed.1001878.s017.docx]

| **Supplemental Table 16. Intercorrelations between changes in food intake 1986-1990: results from the Nurses' Health Study II.** | | | | | | | | | | | | | | | | | | | |
| --- | --- | --- | --- | --- | --- | --- | --- | --- | --- | --- | --- | --- | --- | --- | --- | --- | --- | --- | --- |
|  | Fruit | Vegetables | Juice | Whole-fat dairy | Low-fat dairy | Fried potatoes | Whole grains | Refined grains | Nuts | Sugar sweetened  beverages | Diet beverages | Sweets | Processed meats | Non-processed  meats | Frequency fried food | % *trans* fat | Seafood | Alcohol |  |
| Fruit | -- | **0.29** | **0.13** | 0.03 | **0.12** | -0.03 | **0.13** | 0.06 | 0.05 | -0.02 | -0.01 | 0.01 | 0.01 | 0.01 | -0.03 | **-0.18** | **0.11** | -0.02 |  |
| Vegetables |  | -- | **0.10** | 0.04 | 0.08 | 0.01 | **0.14** | **0.12** | 0.06 | -0.01 | 0.03 | 0.02 | 0.04 | 0.09 | -0.03 | **-0.16** | **0.19** | 0.03 |  |
| Juice |  |  | -- | 0.06 | 0.05 | 0.02 | 0.07 | 0.06 | 0.02 | 0.02 | -0.03 | 0.04 | 0.04 | 0.04 | 0.00 | -0.09 | 0.04 | 0.00 |  |
| Whole-fat dairy |  |  |  | -- | **-0.12** | 0.09 | 0.02 | 0.04 | 0.05 | 0.03 | 0.01 | 0.07 | **0.10** | 0.09 | -0.01 | 0.07 | 0.04 | 0.02 |  |
| Low-fat dairy |  |  |  |  | -- | -0.05 | **0.11** | 0.04 | -0.01 | -0.01 | -0.02 | 0.00 | -0.01 | 0.00 | -0.01 | **-0.14** | 0.03 | -0.03 |  |
| Fried potatoes |  |  |  |  |  | -- | 0.01 | 0.09 | 0.06 | 0.08 | 0.05 | **0.16** | **0.16** | **0.18** | **0.10** | **0.27** | 0.02 | 0.04 |  |
| Whole grains |  |  |  |  |  |  | -- | 0.05 | 0.03 | 0.00 | 0.01 | 0.03 | 0.03 | 0.02 | -0.03 | -0.08 | 0.08 | -0.02 |  |
| Refined grains |  |  |  |  |  |  |  | -- | 0.01 | 0.03 | 0.03 | 0.09 | 0.08 | **0.11** | 0.05 | -0.08 | 0.05 | 0.03 |  |
| Nuts |  |  |  |  |  |  |  |  | -- | 0.02 | 0.02 | 0.07 | 0.04 | 0.04 | 0.01 | 0.00 | 0.04 | 0.02 |  |
| Sugar sweetened beverages | | |  |  |  |  |  |  |  | -- | **-0.16** | 0.06 | 0.05 | 0.06 | 0.03 | -0.07 | 0.00 | 0.00 |  |
| Diet beverages |  |  |  |  |  |  |  |  |  |  | -- | 0.04 | 0.02 | 0.03 | 0.00 | 0.06 | 0.03 | 0.01 |  |
| Sweets |  |  |  |  |  |  |  |  |  |  |  | -- | **0.10** | **0.11** | 0.06 | **0.28** | 0.02 | 0.00 |  |
| Processed meat |  |  |  |  |  |  |  |  |  |  |  |  | -- | **0.20** | 0.05 | **0.13** | 0.03 | 0.03 |  |
| Non-processed meat | |  |  |  |  |  |  |  |  |  |  |  |  | -- | 0.08 | **0.12** | 0.07 | 0.03 |  |
| Frequency fried food | |  |  |  |  |  |  |  |  |  |  |  |  |  | -- | **0.20** | -0.04 | 0.03 |  |
| % *trans* fat |  |  |  |  |  |  |  |  |  |  |  |  |  |  |  | -- | -0.08 | -0.04 |  |
| Seafood |  |  |  |  |  |  |  |  |  |  |  |  |  |  |  |  | -- | 0.04 |  |
| Alcohol |  |  |  |  |  |  |  |  |  |  |  |  |  |  |  |  |  | -- |  |
| Correlations ≥ \|0.10\| (what we considered biologically relevant) are shown in bold. All values shown in bold had a p-value < 0.0001. | | | | | | | | | | | | | | | | |  |  |  |
