# Supplementary material for: Changes in Intake of Fruits and Vegetables and Weight Change in United States Men and Women Followed for Up to 24 Years: Analysis from Three Prospective Cohort Studies
Source: PLoS Med. 2015 Sep 22;12(9):e1001878. doi: 10.1371/journal.pmed.1001878 (PMC4578962; doi:10.1371/journal.pmed.1001878)
Supplement: S17 Table — (DOCX) [file pmed.1001878.s018.docx]

| **Supplemental Table 17. Pearson correlation coefficients (r) between mean consumption of fruits and vegetables estimated by dietary record (DR) and food frequency questionnaire (FFQ) among men in the Health Professionals Follow-up Study [**[**19**](#_ENREF_19)**].** | |
| --- | --- |
|  | **r** |
| Apples, pears | 0.53 |
| Avocados | 0.52 |
| Bananas | 0.76 |
| Blueberries | 0.30 |
| Cantaloupe | 0.40 |
| Watermelon | 0.28 |
| Grapefruit | 0.50 |
| Grapefruit juice | 0.53 |
| Oranges | 0.43 |
| Peaches, plums, apricots | 0.45 |
| Raisins, grapes | 0.42 |
| Strawberries | 0.24 |
| Yams, sweet potatoes | 0.24 |
| Beans, lentils | 0.19 |
| Broccoli | 0.29 |
| Brussels sprouts | 0.31 |
| Sauerkraut | 0.23 |
| Cabbage, coleslaw | 0.21 |
| Cooked cabbage | 0.32 |
| Carrots | 0.34 |
| Cauliflower | 0.20 |
| Celery | 0.19 |
| Corn | 0.32 |
| Eggplant, zucchini | 0.20 |
| Mixed, stir-fry vegetables | 0.13 |
| Peas, lima beans | 0.31 |
| Peppers | 0.38 |
| Spinach | 0.18 |
| Kale, mustard greens | 0.17 |
| Iceberg/romaine lettuce | 0.53 |
| String beans | 0.21 |
| Tofu, soybeans, soy burger, miso, other soy protein | 0.44 |
| Tomatoes | 0.40 |
| Winter squash | 0.28 |
