# Supplementary material for: Changes in Intake of Fruits and Vegetables and Weight Change in United States Men and Women Followed for Up to 24 Years: Analysis from Three Prospective Cohort Studies
Source: PLoS Med. 2015 Sep 22;12(9):e1001878. doi: 10.1371/journal.pmed.1001878 (PMC4578962; doi:10.1371/journal.pmed.1001878)
Supplement: S18 Table — (DOCX) [file pmed.1001878.s019.docx]

| **Supplemental Table 18. Exclusions (sequential) at baseline.** | | | |  |
| --- | --- | --- | --- | --- |
|  |  | **HPFS** | **NHS** | **NHS II** |
| Baseline | | 1986 - 1990 | 1986 - 1990 | 1991 - 1995 |
| Multiple records | | 0 | 0 | 253 |
| Died before data collection | | 1 | 37 | 253 |
| 70+ blank responses on FFQ | | 298 | 0 | 21 |
| Implausible reported energy intake | | 954 | 7,658 | 18,027 |
| Cancer | | 3,488 | 10,556 | 2,524 |
| Diabetes | | 2,197 | 4,509 | 1,746 |
| Ulcerative colitis | | 611 | 1,181 | 1,467 |
| Pulmonary embolism | | 278 | 439 | 2,636 |
| Coronary artery bypass graft | | 2,224 | 422 | 23 |
| Myocardial infarction | | 1,322 | 1,951 | 521 |
| Angina | | 1,107 | 3,621 | 496 |
| Stroke | | 324 | 511 | 402 |
| Lupus |  | 0 | 405 | 339 |
| Irritable bowel | | 27 | 100 | 112 |
| Over age 65 years | | 4,794 | 4,710 | 0 |
| Pregnant | | NA | 0 | 8,994 |
| Missing data | |  |  |  |
|  | Physical activity | 180 | 29,674 | 77 |
|  | Diet | 10,530 | 11,813 | 2,119 |
|  | BMI | 522 | 911 | 422 |
|  | Weight | 112 | 157 | 1,708 |
