# Supplementary material for: Changes in Intake of Fruits and Vegetables and Weight Change in United States Men and Women Followed for Up to 24 Years: Analysis from Three Prospective Cohort Studies
Source: PLoS Med. 2015 Sep 22;12(9):e1001878. doi: 10.1371/journal.pmed.1001878 (PMC4578962; doi:10.1371/journal.pmed.1001878)
Supplement: S19 Table — (DOCX) [file pmed.1001878.s020.docx]

| **Supplemental Table 19. Baseline (mean, SD) fruit and vegetable intake (servings/day) of men and women in three prospective cohorts.** | | | |
| --- | --- | --- | --- |
|  | **HPFS** | **NHS** | **NHS II** |
|  | **n = 19,316** | **n = 40,415** | **n = 73,737** |
|  | **1986** | **1986** | **1991** |
| Melon | 0.13 (0.11) | 0.17 (0.11) | 0.08 (0.09) |
| Citrus Fruits | 0.35 (0.28) | 0.32 (0.19) | 0.19 (0.23) |
| Berries | 0.11 (0.11) | 0.16 (0.11) | 0.16 (0.20) |
| Legumes | 0.31 (0.19) | 0.20 (0.09) | 0.22 (0.22) |
| Cruciferous Vegetables | 0.44 (0.22) | 0.47 (0.18) | 0.41 (0.34) |
| Green Leafy Vegetables | 0.74 (0.37) | 0.83 (0.31) | 0.67 (0.53) |
| Peaches, Plums, Apricots | 0.10 (0.11) | 0.17 (0.11) | 0.12 (0.17) |
| Raisins & Grapes | 0.13 (0.16) | 0.10 (0.10) | 0.10 (0.18) |
| Avocados | 0.03 (0.04) | 0.02 (0.03) | 0.02 (0.05) |
| Bananas | 0.27 (0.21) | 0.24 (0.14) | 0.23 (0.24) |
| Apples & Pears | 0.34 (0.26) | 0.32 (0.18) | 0.29 (0.30) |
| Strawberries | 0.07 (0.07) | 0.11 (0.08) | 0.12 (0.15) |
| Blueberries | 0.04 (0.05) | 0.05 (0.05) | 0.04 (0.08) |
| Prunes | NA | 0.03 (0.08) | 0.02 (0.10) |
| Oranges | 0.23 (0.21) | 0.19 (0.13) | 0.12 (0.16) |
| Grapefruit | 0.19 (0.22) | 0.21 (0.18) | 0.11 (0.22) |
| String Beans | 0.15 (0.09) | 0.18 (0.08) | 0.15 (0.15) |
| Broccoli | 0.17 (0.10) | 0.21 (0.09) | 0.21 (0.18) |
| Cabbage | 0.11 (0.09) | 0.10 (0.06) | 0.08 (0.10) |
| Cauliflower | 0.10 (0.08) | 0.11 (0.07) | 0.10 (0.13) |
| Brussels Sprouts | 0.04 (0.05) | 0.03 (0.04) | 0.03 (0.06) |
| Carrots | 0.22 (0.19) | 0.33 (0.18) | 0.35 (0.38) |
| Corn | 0.15 (0.09) | 0.13 (0.06) | 0.16 (0.14) |
| Peas | 0.15 (0.09) | 0.13 (0.06) | 0.12 (0.12) |
| Mixed Vegetables | 0.12 (0.10) | 0.07 (0.06) | 0.09 (0.13) |
| Beans | 0.10 (0.09) | 0.07 (0.05) | 0.09 (0.12) |
| Celery | 0.15 (0.15) | 0.20 (0.14) | 0.16 (0.22) |
| Winter Squash | 0.05 (0.05) | 0.06 (0.04) | 0.03 (0.06) |
| Summer Squash | 0.07 (0.07) | 0.09 (0.06) | 0.07 (0.11) |
| Peppers | 0.13 (0.13) | 0.17 (0.12) | 0.14 (0.19) |
| Tomatoes | 0.36 (0.21) | 0.38 (0.17) | 0.26 (0.25) |
| Tofu & Soy | 0.02 (0.05) | 0.01 (0.03) | 0.01 (0.08) |
| Onions | NA | NA | 0.34 (0.38) |
| Potatoes* | 0.34 (0.17) | 0.35 (0.13) | 0.29 (0.20) |
| *Baked, boiled, or mashed white potatoes, sweet potatoes and yams; excludes french fries and potato chips. | | | |
| NA = not on baseline FFQ. |  |  |  |
