# Supplementary material for: Changes in Intake of Fruits and Vegetables and Weight Change in United States Men and Women Followed for Up to 24 Years: Analysis from Three Prospective Cohort Studies
Source: PLoS Med. 2015 Sep 22;12(9):e1001878. doi: 10.1371/journal.pmed.1001878 (PMC4578962; doi:10.1371/journal.pmed.1001878)
Supplement: S2 Text — (DOC) [file pmed.1001878.s022.doc]

**Title:** Fiber content and glycemic load of fruits, vegetables and whole grains and weight change over time

**Authors:** Monica Bertoia, Ken Mukamal, Leah Cahill, Tao Hao, David Ludwig, Dariush Mozaffarian, Walter Willett, Frank Hu, Eric Rimm

**Purpose:** To examine whether the fiber content and glycemic load (GL) of specific fruits, vegetables and whole grains are associated with weight change.

**Background:** A recent study using NHS, NHS II, and HPFS data found that increased fruit, vegetable and whole grain consumption was associated with weight loss over 4 years in men and women. Higher fiber intake increases satiety, which in turn reduces total energy intake and weight gain. Also, lower GL foods produce fewer and smaller postprandial glucose spikes which theoretically decrease hunger and reduce fat storage. We will focus our analysis on which particular fruits, vegetables and whole grains have the largest impact on weight change. We hypothesize that higher fiber, lower GL fruits, vegetables and whole grains are more strongly associated with beneficial weight change than lower fiber, higher GL foods. The results from this analysis may help to improve previous dietary recommendations and provide individuals with better food based guidance. Furthermore, our results will contribute to current knowledge and strategies for obesity prevention.

**Study population:**

Men and women from three large US cohorts:

1. the Health Professionals Follow-up Study (HPFS) cohort of 51,529 male health professionals from all 50 states enrolled in 1986 (first FFQ in 1986)
2. the Nurses’ Health Study (NHS) cohort of 121,701 registered nurses from 11 US states enrolled in 1976 (first FFQ in 1986)
3. the Nurses’ Health Study II (NHS II) cohort of 116,686 younger female registered nurses from 14 states enrolled in 1989 (first FFQ in 1991)

Exclusion criteria:

- - obesity, diabetes, cancer, CVD, renal disease, pulmonary disease, or liver disease at baseline
  - censor new cases of disease 6 years prior to diagnosis
  - missing baseline lifestyle habits data
  - implausible reported dietary energy intake (<600 or >3500 kcal/day for women or <800 or >4200 kcal/day for men), blank responses for more than nine items on the FFQ
  - newly pregnant or lactating (women only) at baseline and temporarily exclude during follow-up – add back in at next FFQ
  - over age 65 years (age-related loss of lean muscle mass) at baseline and censor during follow-up

**Exposure:**

Increased intake of fruits and vegetables will be defined as an increase in number of servings per day. Increased intake of whole grains will be defined as an increase in total grams/day. We will model intake at baseline of interval as well as change during interval.

We will categorize fruits and vegetables several different ways, first as groups and then as individual foods:

1. by scientific class (ex: citrus fruits)
2. high or low GL (median)
3. high or low fiber (median)

We will categorize whole grains as grams/day of:

1. high or low GL (median)
2. high or low fiber (median)
3. by type of cereal grain (ex: wheat, oat, corn)
4. by type of whole grain (intact/milled or not)

We will additionally look at change in tertiles and explore at a joint category analysis using medians (high fiber low GL, high fiber high GL, low fiber low GL, and low fiber low GL)

**Outcome:**

Weight change within each 4-year period measured in absolute units (pounds) and relative units (percent). There are 5 4-year periods in the NHS (over 20 years), 4 in the NHS II (16 years), and 5 in the HPFS (20 years).

**Covariates:**

Age, baseline BMI for that particular 4-year period, physical activity, TV watching, alcohol, sleep duration, smoking, blood pressure, post-menopausal status, hormone therapy use, medication use, and other dietary factors (sugar-sweetened beverages, french fries, potato chips, potatoes, unprocessed red meat, processed meats, nuts, yogurt). Total energy intake will not be included as a covariate because it is potentially on the causal pathway. Height, region, marital status. These are change variables (not absolute levels) except for age, baseline BMI for time period, and sleep which cannot be updated.

**Analyses:**

- We will assess the independent relationships between changes in intake of high fiber fruits, vegetables and whole grains and low GL fruits, vegetables and whole grains and weight changes within 4-year periods by including all lifestyle and dietary factors simultaneously.
- We will include common types of fruits and vegetables in a model simultaneously.
- We will use multivariable linear regression with robust variance to account for within-individual repeated measures (PROC GENMOD with REPEATED statement).
- We will consider nonlinear effects by using indicator variables and effect modification by using interaction terms.
- We will consider censoring weight change at the 0.5^th^ and 99.5^th^ percentiles to minimize the influence of outliers.
- For missing covariate data, we will use missing indicators for categorical variables and carry the last observation forward for continuous variables.
- Sensitivity analyses among nonsmokers, stratified by age, baseline BMI, physical activity, diabetes/insulin, etc.
- We will pool the data using an inverse variance weighted fixed effects meta-analysis
